# Copy number and sequence variation in ribosomal DNA and the transposon, *Pokey*, in mutation accumulation lines of *Daphnia obtusa*

**DOI:** 10.1093/g3journal/jkaf279

**Published:** 2025-11-20

**Authors:** Abir Elguweidi, Teresa J Crease

**Affiliations:** Department of Integrative Biology, University of Guelph, Guelph, ON, N1G 2W1, Canada; Department of Animal Science, University of Benghazi, Benghazi 21861, Libya; Department of Integrative Biology, University of Guelph, Guelph, ON, N1G 2W1, Canada

**Keywords:** mutation accumulation lines, *Daphnia obtusa*, ribosomal DNA, copy number variation, haplotype variation, recombination rate, *Pokey* transposons

## Abstract

Ribosomal DNA (rDNA) occurs as tandem arrays of a repeat unit containing the genes encoding *18S*, 5.8S, and *28S* rRNA separated by spacers. These rRNAs form the catalytic core of ribosomes and thus play a crucial role in protein synthesis. Due to its repetitive nature, rDNA copy number varies within and between eukaryotic species through recombination, which also results in homogenization of repeat sequences within species (concerted evolution). However, the recombination rate within rDNA has not been extensively estimated. Despite concerted evolution and strong selection to maintain the sequence of rRNA genes, some transposons insert into specific sequences in the *28S* gene. We used short-read whole-genome sequences to examine the dynamics of change in rDNA copy number and sequence variation in 90 samples from clonally propagated *Daphnia obtusa* mutation accumulation (MA) lines over ∼95 generations. We also tracked the number of *Pokey* elements, a DNA transposon that inserts into the *28S* gene of species in the subgenus *Daphnia*. We observed an overall decline in rDNA copy number across MA lines between generations 5 and ∼87, although both increases and decreases were observed over short intervals. The diploid *28S* copy number ranged from 144 to 1,274, with a mean of 425.2. Diploid *Pokey* number varied from 65 to 537 and was significantly positively correlated with *28S* copy number. Moreover, the element persisted in all lines even after large reductions in *28S* copy number. We found that estimating rates of rDNA copy number change over long intervals resulted in substantial underestimates, as shorter intervals revealed that large copy number changes could occur in as few as 5 generations. We identified 5 rDNA haplotypes based on 58 single nucleotide polymorphisms (SNPs) that were distributed across the *18S* and *28S* genes, and the 3 non-repetitive intergenic spacer regions. We also identified 6 *Pokey* haplotypes based on 113 SNPs. The number of these haplotypes was strongly correlated with the number of the 3 most common rDNA haplotypes. By tracking changes in haplotype frequency and copy number within 4 MA lines over short time intervals, we estimated the mean rDNA recombination rate to be 0.094 events/generation. These results reveal that rapid changes can occur in rDNA over short timescales and show that *Pokey* transposon dynamics are tightly linked to rDNA structure.

## Introduction

Ribosomal DNA (rDNA) in eukaryotes consists of a multigene family organized into tandemly repeated units each containing 1 copy of the *18S*, 5.8S, and *28S* rRNA genes, separated by intergenic spacers (IGSs). These repeated units form nucleolar organizing regions and can be located on 1 or more chromosomes (Long and Dawid 1980). rDNA copies typically exhibit a high degree of sequence similarity within a species despite divergence between species, which is known as concerted evolution (Zimmer et al. 1980). Concerted evolution is driven by recombination occurring both within and between rDNA arrays (Dover 1982; Eickbush and Eickbush 2007).

DNA copy number in eukaryotic genomes can range from tens to tens of thousands (Prokopowich et al. 2003). Due to their repetitive structure, rDNA arrays are prone to expansion and contraction through unequal recombination events, particularly sister chromatid exchange (SCE) during both meiosis and mitosis, as well as homologous (interchromosomal) chromatid exchange (HCE) (Motovali-Bashi et al. 2004). Unequal recombination involves exchanges between misaligned chromatids, causing 1 chromatid to gain additional rDNA copies at the expense of the other (Tartof 1974). Empirical evidence suggests that SCE is the predominant form of recombination in rDNA arrays, occurring more frequently than HCE (Petes 1980; Seperack et al. 1988; Schlötterer and Tautz 1994; Zhang et al. 2008). These mechanisms drive considerable variation in rDNA copy number both within and between species of eukaryotic organisms (e.g. Zhang et al. 1990; Gibbons et al. 2014; McElroy et al. 2021; Elguweidi and Crease 2024).

Recombination in rDNA causes both increases and decreases in total copy number. Copy number reductions below a threshold value can cause detrimental effects. For example, reduced rDNA copy number in *Drosophila* can result in defects in ribosome biogenesis, causing delayed development and visible phenotypes like bobbed (*bb*), where the bristles on the abdomen are extremely short and deformed (Hawley and Marcus 1989). More recently, Paredes and Maggert (2009) showed that reductions in rDNA copy number on the *Drosophila* Y chromosome decrease gene silencing in other genomic regions, with larger deletions producing stronger effects. Such changes in copy number can influence genome-wide expression by disrupting the balance between heterochromatin and euchromatin during development (Paredes et al. 2011).

On the other hand, deficiencies in rDNA copy number can be compensated through rDNA magnification, which restores lost repeats and ensures proper cellular function. For example, Kindelay and Maggert (2024) highlighted the dynamic nature of rDNA arrays in *Drosophila*, which undergo both losses and compensatory magnification that can affect genome stability and nuclear organization. rDNA amplification can also compensate for deficiencies elsewhere in the genome. For example, in *Saccharomyces cerevisiae* mutants lacking RNA polymerase I subunits, an increase in rDNA repeats allows survival by enabling transcription using RNA polymerase II. These mutants can only survive after the number of rDNA repeats has increased (Oakes et al. 1999; Vu et al. 1999). Conversely, higher rDNA copy number has been associated with markers of systemic inflammation and reduced renal function in humans (Rodriguez-Algarra et al. 2025) and an increased risk of lung cancer (Hosgood et al. 2019).

Despite its potentially beneficial effects, excessive recombination, especially SCE, can be detrimental as it may lead to the accumulation of extrachromosomal rDNA circles (ERCs; Sinclair and Guarente 1997), which have been linked to aging, particularly in *S. cerevisiae* (Nelson et al. 2019). Similarly, Valori et al. (2020) found that rDNA arrays in metastatic breast cancer cells were more unstable than arrays in healthy tissue leading to both loss and gain of rDNA repeats. Moreover, recombination events involving DNA double-strand breaks (DSBs) can cause mutations, loss of heterozygosity, and chromosome rearrangements (Cannan and Pederson 2016).

Although extremes of rDNA copy number can have detrimental phenotypic effects, variation within the natural range appears to be effectively neutral under standard conditions. For example, only ∼35 rDNA copies are required for basic ribosome function in yeast, yet natural strains typically carry ∼90 to 300 copies, and small fluctuations within this range often do not affect fitness (Thornton et al. 2025). Similarly, large differences in *C. elegans* rDNA copy number (∼130 vs ∼417 copies) show no measurable impact on rRNA abundance, competitive fitness, early-life fertility, lifespan, or global transcription under standard laboratory conditions (Hall et al. 2025) suggesting that such variation is neutral or nearly neutral. Despite the observation that rDNA copy number is highly variable in natural populations, the specific rDNA copy number thresholds underlying developmental and tissue-specific phenotypes, as well as the potential fitness consequences of extremely high rDNA copy number, are not well understood in most multicellular organisms. Moreover, the rate of rDNA recombination has only been estimated in a few organisms such as *S. cerevisiae* (0.001 events/generation, Szostak and Wu 1980; 0.0013, Merker and Klein 2002), *Drosophila* (0.0001, Williams et al. 1989), murine cell lines (0.00001, Nelson et al. 1989), and *Daphnia obtusa* mutation accumulation (MA) lines (0.074, McTaggart et al. 2007).

*Daphnia*, a freshwater zooplankter in the crustacean order Cladocera, has been widely used in ecological, developmental, and ecotoxicological research, making it an important model organism in these fields (Reynolds 2011; Ebert 2022). *Daphnia* play a key role in aquatic ecosystems by grazing on phytoplankton and serving as essential prey for fish and other predators (Miner et al. 2012). They typically reproduce through cyclical parthenogenesis in which direct-developing eggs are produced via apomixis during favorable conditions. However, diapausing eggs are produced via meiosis when conditions become unfavorable, such as drought or freezing (Ebert 2022). *D. obtusa* is a species commonly found in temporary ponds across the midwestern United States and southern Ontario (Schwartz et al. 1985; Innes 1989; Hebert and Finston 1996). It is in the same subgenus (*Daphnia*) as *Daphnia pulex*, which, along with *Daphnia magna* (subgenus: Ctenodaphnia), are the most studied *Daphnia* species (Ebert 2022).

MA experiments coupled with whole-genome sequencing have proven to be an effective method for studying genome-wide mutation patterns (Keith et al. 2016). Repeated bottlenecking of genetically identical lines in MA experiments minimizes the efficiency of natural selection, allowing most new mutations to be captured. The only exceptions are mutations that result in immediate lethality or sterility (Kibota and Lynch 1996). One advantage of using *Daphnia* in MA experiments is that they produce direct-developing eggs clonally, which eliminates the need to create highly inbred lines from wild-caught individuals.

Several studies have used the same set of *D. pulex* MA lines that were exposed to copper and nickel to investigate the impact of these metals on the *D. pulex* genome. Chain et al. (2019) investigated rates of large-scale deletions and duplications, while Bull et al. (2019) estimated single nucleotide mutation rates. Harvey et al. (2020) assessed changes in the number of *18S* genes using quantitative (q)PCR and found a tendency for copy number to decrease over time with metal exposure. In addition, Elguweidi et al. (2025) used whole-genome sequencing to study the rate of rDNA copy number change and sequence variation in the same *D. pulex* MA lines. Although the results indicated that metal exposure does not directly impact rDNA copy number, it may indirectly influence copy number by altering recombination rates.

McTaggart et al. (2007) studied rDNA variation in clonally propagated MA lines of *D. obtusa* descended from a single wild-caught female. They estimated rDNA recombination rate to be 0.02 to 0.06 events per generation by analyzing changes in the frequency of length variants in an expansion segment of the *18S* gene in 4 MA lines every 5 generations. Subsequently, LeRiche et al. (2014) used qPCR to track changes in *18S* and *28S* copy number in the same 4 lines analyzed by McTaggart et al. (2007), as well as 20 lines after approximately 87 generations. Despite considerable variation among lines, the average rate of copy number change was <1 gene per generation after 87 generations indicating no strong bias in the direction of change.

In addition to recombination-based mechanisms, transposable elements (TEs) can also influence rDNA dynamics. For example, the non-LTR R-element family inserts into specific sites in the *18S* or *28S* rRNA gene, which makes the gene nonfunctional. In addition, the *R2* element can induce DSBs that can drive rDNA magnification via unequal SCE (Eickbush and Eickbush 2015; Jakšić et al. 2017). Some R-elements are ancient; for example, *R2* is found in phylogenetically diverse metazoans and is estimated to have originated before the metazoan Cambrian explosion, 850 to 600 million years ago (Kojima et al. 2006). Moreover, *R2* elements have co-evolved with rDNA arrays in metazoans for millions of years (Malik et al. 1999; Eickbush and Eickbush 2007; Eickbush and Eickbush 2015). Plants harbor analogous systems; for example, LTR retrotransposons like Ty1-*copia* in *Arabidopsis* rDNA generate structural variation (Neumann et al. 2019). These interactions suggest that TE–rDNA relationships range from parasitic to mutualistic, as TEs may stabilize arrays by suppressing recombination (Guerrero and Maggert 2011).

Although most rDNA-specific TEs are retrotransposons, the DNA transposon *Pokey* inserts into a specific TTAA site in the *28S* rDNA of *Daphnia* (Penton and Crease 2004), although it can also be found outside rDNA at TTAA sites that are similar to the *28S* insertion site (Eagle and Crease 2012; Elliott et al. 2013). *Pokey* belongs to the *piggy-Bac* superfamily of DNA TEs, which has been found in organisms including fungi, plants, insects, crustaceans, urochordates, amphibians, fishes, and mammals (Sarkar et al. 2003). However, to the best of our knowledge, other *piggyBac*-like elements have not been found in rDNA. The relative distribution of *Pokey* inside and outside rDNA varies among *Daphnia* species; it is primarily found outside rDNA in *D. pulex* (Eagle and Crease 2012, 2016) but is found almost entirely inside rDNA in *D. obtusa* (LeRiche et al. 2014). Moreover, *Pokey* copy number is strongly correlated with *28S* copy number in *D. obtusa*.

We used short-read whole-genome sequencing to track changes in rDNA and *Pokey* variation in the *D. obtusa* MA lines studied by McTaggart et al. (2007) and LeRiche et al. (2014). We used this data to estimate rates of rDNA copy number change, identify sequence haplotypes, and estimate rates of rDNA recombination-based on changes in haplotype frequency and copy number. We also compared copy number estimates based on short-read sequencing with those obtained via qPCR, as well as recombination rates based on short indel variants in an expansion segment of the *18S* gene with those based on single nucleotide polymorphisms (SNPs) across most of the rDNA repeat.

## Methods

### *D. obtusa* MA lines

The *D. obtusa* MA lines analyzed in this study were originally described by McTaggart et al. (2007) and LeRiche et al. (2014). In brief, MA lines were established in 2001 from the clonal offspring of a single female collected from a pond in Trelease Woods near Urbana, Illinois (40.128504, −88.126030). Fifty MA lines were derived from these offspring and propagated apomictically. One daughter was randomly selected each generation to continue the line. Laboratory conditions were controlled to promote clonal reproduction and avoid production of diapausing eggs. Detailed methods on the propagation of these MA lines are provided in McTaggart et al. (2007).

McTaggart et al. (2007) sampled lines at generation 5 (MA05 samples) and again at ∼generation 87 (MA87 samples). Four lines (3, 12, 29, and 30) were sampled approximately every 5 generations up to generation 95 and are referred to as the “fine-grained” (FG) lines. Each MA sample consists of a pool of the siblings of the female selected to continue the line and is designated by its line and generation number (e.g. 02-89 indicates line 2 sampled at generation 89).

McTaggart et al. used the CTAB method (Doyle and Doyle 1987) to extract DNA from the pool of the siblings. Ninety of these DNA extractions still contained sufficient DNA for short-read genome sequencing and included 43 samples from the 4 FG lines taken between generation 10 and 92 (12 samples from line 3, 9 samples from line 12, 16 samples from line 29, 6 samples from line 30), 26 samples taken at generation 5 (MA05 samples), and 36 samples taken at ∼generation 87 (MA87 samples, including samples from the FG lines). Sixteen lines were sampled at both generation 5 and generation ∼87 and are referred to as the paired lines. One sequence library was prepared for each sample by the AAC Genomics Facility at the University of Guelph using the Illumina DNA Library Prep kit (Illumina, Inc.). In addition, a second library was prepared from the DNA extract of sample 29-85 (29-85b) for a total of 91 sequenced genomes. Four replicates of each library, including the duplicates were sequenced using 100 bp paired-end reads on a NovaSeq 6000 (Illumina, Inc.) at the CRLB-GMEL facility in Hamilton, Ontario, Canada. The average number of reads per sample was 92.2 million with a minimum of 19.1 million and a maximum of 241.7 million. All sequences have been archived in the NCBI Sequence Read Archive under BioProject PRJNA1204324 (Crease 2024).

Adapters were trimmed from the sequences with Trimmomatic version 0.39 (Bolger et al. 2014). BWA-MEM version 0.7.17 (Li and Durbin 2009) was then used to align the cleaned sequences to reference sequences of 5 rDNA regions, 2 regions of a *Pokey* element and 16 exons from single-copy genes (SCG). The resulting SAM files were converted into sorted BAM files using Samtools version 1.7 (Danecek et al. 2021 ). Duplicate sequences were removed with Picard version 2.23.2 (Broad Institute 2019) using default settings. Bedtools version 2.30.0 (Quinlan and Hall 2010) was used to calculate the per-base read depth for each reference sequence in the deduplicated BAM files. Finally, a summary of the read depth statistics (mean, mode, and median) was generated using stats2.sh (McElroy et al. 2021).

### Reference sequences

The rDNA repeat was divided into 5 regions: 2 rRNA genes, *18S* (2,292 bp) and *28S* (4,376 bp), and 3 unique IGS regions (Ambrose and Crease 2010). The sequence between the *18S* and *28S* genes, which contains the *5.8S* gene and the internal transcribed spacers, is not available for *D. obtusa*, so this region was not included in our analysis. In addition, the subrepeats between IGS unique regions were excluded from all analyses as it was not possible to differentiate them from each other using short-read sequence data. Sequences were obtained from GenBank and included the *D. pulex 18S* gene (accession AF014011; Crease and Colbourne 1998), the *Daphnia pulicaria 28S* gene (accession AF346514; Omilian and Taylor 2001), and a *D. obtusa* IGS sequence (accession EU595564; Ambrose and Crease 2010). In addition, the sequence of a *Pokey* element (accession PV424089) cloned from a *D. obtusa* isolate collected from a pond near Natchez, Tennessee, United States, was divided into 2 regions: the putative transposase gene (1,468 bp) and the sequence downstream of the transposase gene (1,224 bp). The 5′ region of *Pokey* elements in *Daphnia* is highly variable in sequence and length due to the presence of subrepeats (Penton et al. 2002; Elliott et al. 2013). Thus, we did not map the *Daphnia* genome sequences to the *Pokey* sequence upstream of the transposase gene. Finally, 16 exons from SCGs were used to estimate the haploid rDNA and *Pokey* copy number. We chose the same exons used by Elguweidi and Crease (2024) to estimate rDNA copy number in *D. pulex* and then obtained *D. obtusa* sequences for them by searching the *D. obtusa* reference genome sequence (accession GCA_016170125.1). All reference sequences are provided in Supplementary File 1.

### Estimating haploid rDNA and Pokey copy number

The genome sequences were aligned to the reference sequences for the 5 rDNA regions, 2 *Pokey* regions, and 16 exons as described above. We averaged the read depth of each region in the 4 replicate libraries as they were very similar to each other. To estimate the haploid copy number of each rDNA and *Pokey* region, we divided the mean read depth of each region by the mean read depth of the 16 exons. The diploid copy number of each rDNA and *Pokey* region was then obtained by multiplying the haploid estimate by 2. We performed a regression analysis in R (R Core Team 2020) to assess the correlation between rDNA and *Pokey* copy numbers and to determine the slope of the best-fit line, which is expected to be 1 for the rDNA regions if each rDNA repeat contains 1 copy of each.

We quantified the rate of diploid copy number change per generation (change rate) in the *28S* gene by dividing the difference in copy number between 2 samples by the number of generations between them (the interval). We did this for all pairs of samples in the FG lines (i.e. 03-05 vs each of 03-15, 03-30, 03-35, up to 03-85; 03-15 vs 03-30, 03-35, up to 03-85 and so on for all samples in a line). This provided change rate estimates based on many interval sizes throughout the course of the MA experiment. For example, the change rates between 03-05 and 03-15 and between 03-75 and 03-85 both provide estimates for an interval of ten generations. However, the first comparison occurs after only 15 generations of propagation while the other occurs after 85 generations. We also estimated the change rate between the 2 samples in each of the 16 paired lines sampled at both generation 5 and ∼ generation 87. Finally, we used the median value of *28S* copy number in the MA05 samples as an estimate of copy number in the progenitor female and then used it to calculate the change rate between generation 0 and every sample. To assess the magnitude of change, regardless of direction, we also calculated the absolute value of each change rate.

### Variant calling

We used Bcftools version 1.11 (Li 2011) to detect SNPs within rDNA and *Pokey* and to create an mpileup file for each genome. The resulting mpileup files were then processed using R to generate summaries of read depth for each of the 4 nucleotides at every variable nucleotide position. Allele counts below 5 at any nucleotide within a sample were adjusted to 0, and the total read depth for that site was recalculated accordingly. SNPs with a total read depth of <99 within a sample were excluded. Allele frequencies for each SNP in each sample were calculated by dividing the nucleotide's read count by the total read depth across all 4 nucleotides. SNPs were removed from further analysis if the mean frequency of allele 1 (the reference allele) was >0.990 across all 91 samples. We used the secondary structure of the *D. pulex 18S* gene (Crease and Colbourne 1998) to determine the location of SNPs in this gene. To locate SNPs in the *28S* gene, we aligned the *D. pulicaria 28S* sequence with the *Drosophila melanogaster* sequence, whose secondary structure was characterized by Wuyts et al. (2001).

### Haplotype estimation

We used the R package haploSep (Pelizzola et al. 2021) to detect rDNA haplotypes and estimate their frequencies from the SNP data within each sample. Before running haploSep, we used haploSelect to estimate the expected number of haplotypes. If this estimate exceeded the logarithm base 2 of the total SNP count, we followed the recommendation of Pelizzola et al. (2021) to use the smaller value in haploSep. In addition, we set the “bias” parameter to FALSE to ensure that haplotype frequencies within each sample summed to 1. This setting may have excluded minor haplotypes, especially those with low-frequency alleles, from detection. However, applying these settings produced identical results across multiple runs of haploSep. Haplotype frequencies were converted to haplotype numbers within samples by multiplying the frequencies by the diploid *28S* copy number. We used the diploid number of the *Pokey* 3′ end to estimate the copy number of *Pokey* haplotypes.

To explore the relationship between rDNA copy number and haplotype variation, we first estimated the expected heterozygosity (H_e_) for each sample using the formula 1 − (∑p_i_²), where p_i_ is the frequency of the ith haplotype in the sample. We then performed a linear regression analysis in R to evaluate the relationship between H_e_ and the diploid *28S* copy number. We also calculated H_e_ for the *Pokey* haplotypes separately.

We generated haplotype networks based on the number of nucleotide differences at the rDNA and *Pokey* SNP loci across the 91 samples using the Templeton, Crandall, and Sing (TCS) method (Templeton et al. 1992) and the program PopArt (https://popart.maths.otago.ac.nz/).

### Recombination rate of rDNA

We used the method of McTaggart et al. (2007) to estimate rDNA recombination rate in the FG lines by comparing changes in diploid haplotype numbers between consecutive samples. This method is based on the assumption that changes in haplotype number are a result of unequal recombination between chromatids or homologous chromosomes, which can affect the copy number of 1 or more haplotypes. To do this, we applied the G-test of independence and calculated the residuals for each haplotype. The G-test is a conservative approach that identifies differences in the relative frequency across all haplotypes between 2 samples, so we also used the residuals to detect changes in individual haplotypes that might not result in a significant G-test. The G-test formula was based on Woolf (1957): *G* = 2∑ [*O_i_* ×ln (*O_i_*/*E_i_*)], where *O_i_* is the observed number of the *i*th haplotype and *E_i_* is the expected number of the *i*th haplotype in each cell of the 2 (samples)×5 (haplotypes) contingency table. The expected value for each cell of the contingency table was calculated as [(row total × column total)/grand total]. We corrected the *P*-values across all 38 tests in the 4 FG lines using the Benjamini and Hochberg (1993) method in R. Each time interval with a significant *G*-value was counted as 1 recombination event.

The residuals for each haplotype in each sample were calculated as [(*O_i_*−*E_i_*)/√*E_i_*] (McTaggart et al. 2007). We considered any residual values >2 or <−2 to indicate a substantial change in the haplotype number during the time interval. Therefore, if at least 1 residual value within a time interval was outside the range −2 to 2, 1 recombination event was counted, even if the overall G-test was not significant.

We also counted a recombination event if there was a significant change in *28S* copy number between consecutive samples. As above, changes in rDNA copy number are assumed to be caused by unequal recombination events. Depending on the distribution of haplotypes along the chromatids, it is possible for total copy number to change without significantly changing the relative frequency of haplotypes. To do this, we calculated the mean and variance of diploid *28S* copy number estimates based on the 16 exons and then used *t*-tests to determine if there was a significant difference in copy number between consecutive samples. We corrected the *P*-values across all 38 tests in the 4 FG lines using the Benjamini and Hochberg (1993) method in R.

To avoid inflating the recombination rate, we only counted 1 event per interval if any one of the following was true for a pair of consecutive samples: the G-test was significant and/or any residual values were outside the range −2 to 2 and/or the difference in *28S* copy number was significant. We calculated the recombination rate per interval as the number of events (1 or 0) divided by the number of generations between the 2 samples. We averaged these rates across all comparisons to estimate the recombination rate for each line and across the 4 FG lines.

## Results

### rDNA copy number

**Fig. 1.**
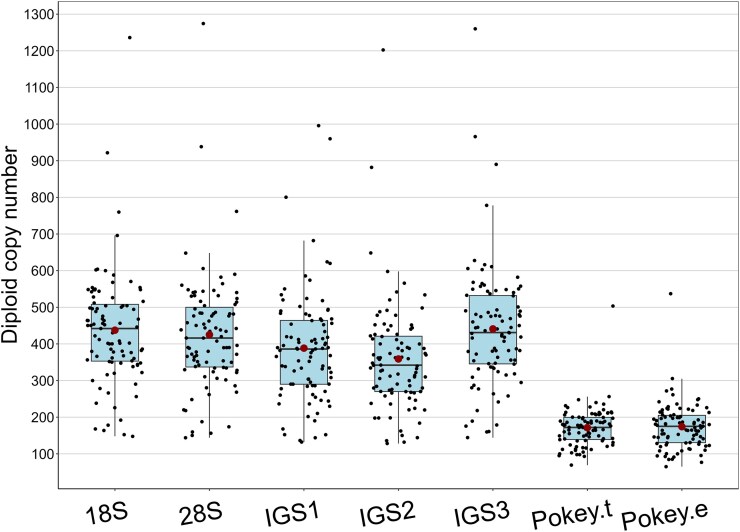
Diploid copy number of 5 rDNA and 2 *Pokey* transposon regions in *D. obtusa* MA lines. Each box represents the interquartile range (IQR), with the line inside the box indicating the median. The whiskers extend to 1.5 times the IQR, and individual data points are shown as dots. The large red dots denote the mean values for each rDNA region. *Pokey*.t refers to the *Pokey* transposase gene. *Pokey*.e refers to the 3′ end of the *Pokey* element.

**Fig. 2.**
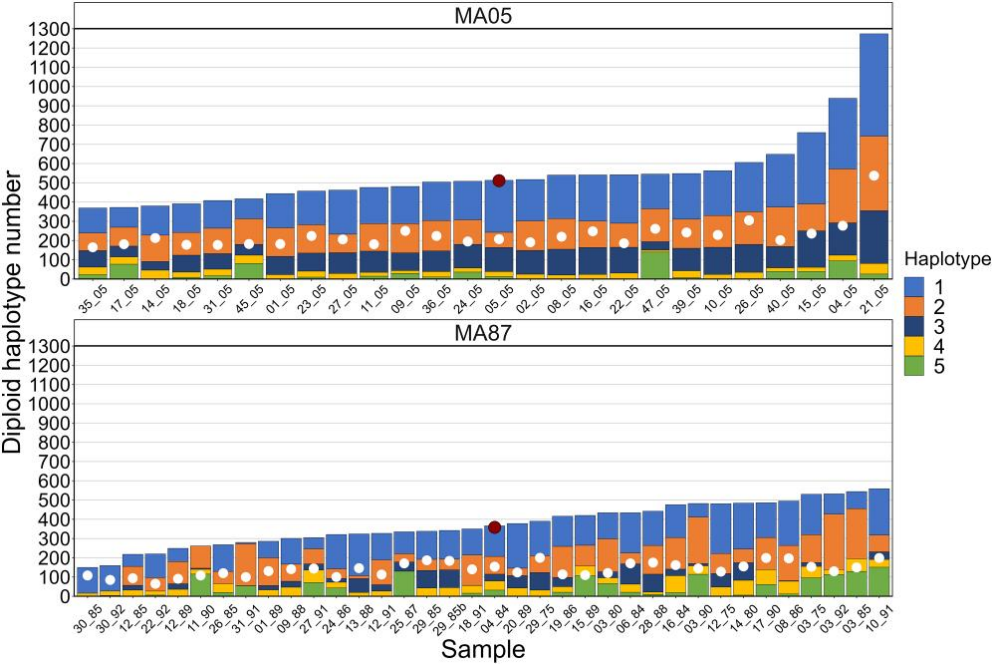
Distribution of 5 rDNA haplotypes in *D. obtusa* MA lines sampled at generation 5 (MA05) and generation ∼87 (MA87). Total diploid copy number is based on the *28S* gene, and the samples are ordered according to the total copy number. The red dot indicates the median copy number for each group. The white dots represent the diploid copy number of the *Pokey* 3′ end.

The diploid copy number of the 5 rDNA regions varied by an order of magnitude across all samples in fewer than 100 generations. The *18S* gene copy number ranged from 148 to 1,236 (mean = 437.4), while the *28S* copy number ranged from 144 to 1,274 (mean = 425.2) (Fig. 1; Supplementary Table 1 in Supplementary File 2). As expected, copy number in the IGS regions was similar to the genes, although the mean and median were lower for IGS1 and IGS2 compared to the genes (Fig. 1). All regions showed a general trend of decreasing copy number between generation 5 (MA05 samples) and generation 75 to 95 (MA87 samples; Supplementary Table 2 in Supplementary File 2). For example, the median value of the *28S* gene was 512 in the MA05 samples (Fig. 2), which declined to 358 in the MA87 samples (Fig. 2). Moreover, a sample taken at generation 5 (21-05) had the highest *28S* copy number (1,274), while the highest copy number in the MA87 samples was 560 (10-91). The duplicate samples (29-85 and 29-85b) showed very similar copy numbers for all rDNA regions (e.g. 338 and 342 for the *28S* gene; Fig. 2; Supplementary Table 1 in Supplementary File 2).

Regression analysis revealed a highly significant relationship between *28S* and *18S* copy numbers (*R*² = 0.996; *P* < 0.00001; Supplementary Fig. 1 in Supplementary File 3), with a regression coefficient close to the expected value of 1 (1.02). Similarly, strong positive correlations were found between the copy numbers of the IGS regions (Supplementary Fig. 1 in Supplementary File 3) with regression coefficients close to 1. The regression coefficients for the spacers in relation to the genes were also close to 1, with the lowest coefficient observed between IGS1 and *28S* at 0.92 (Supplementary Fig. 1 in Supplementary File 3) and the highest coefficient (1.06) observed between IGS3 compared to the *18S* gene.

The rate of diploid *28S* copy number change per generation (change rate) in the FG lines, based on pairwise comparisons of all samples in a line, ranged from −26.40 to 40 with an overall mean of 0.52 across intervals from 2 to 81 generations (Supplementary Table 3 in Supplementary File 2, Supplementary Fig. 2 in Supplementary File 3). The mean change rate in the FG lines was very close to 0 for all intervals except 5 generations, where the mean was 4.57. Of the 29 estimates for this interval, 11 were negative and 18 were positive. This is similar to the MA05 samples, in which change rates ranged from −28.80 to 152.40 with a mean of 6.88 based on a copy number of 512 at generation 0 (Supplementary Table 1 in Supplementary File 2). Thirteen of the 26 estimates were positive as expected given that we used the median value as the progenitor female's copy number. In contrast, change rate in the paired lines ranged from −7.24 to 1.39 with a mean of −1.91 across intervals from 79 to 87 generations (Supplementary Table 3 in Supplementary File 2, Supplementary Fig. 2 in Supplementary File 3). Only 2 of the 16 estimates were positive. This is consistent with results from all the MA87 samples where change rates varied from −4.26 to 0.53 with a mean of −1.60 based on a copy number of 512 at generation 0 (Supplementary Table 1 in Supplementary File 2). Of the 36 estimates, only 4 were positive.

**Fig. 3.**
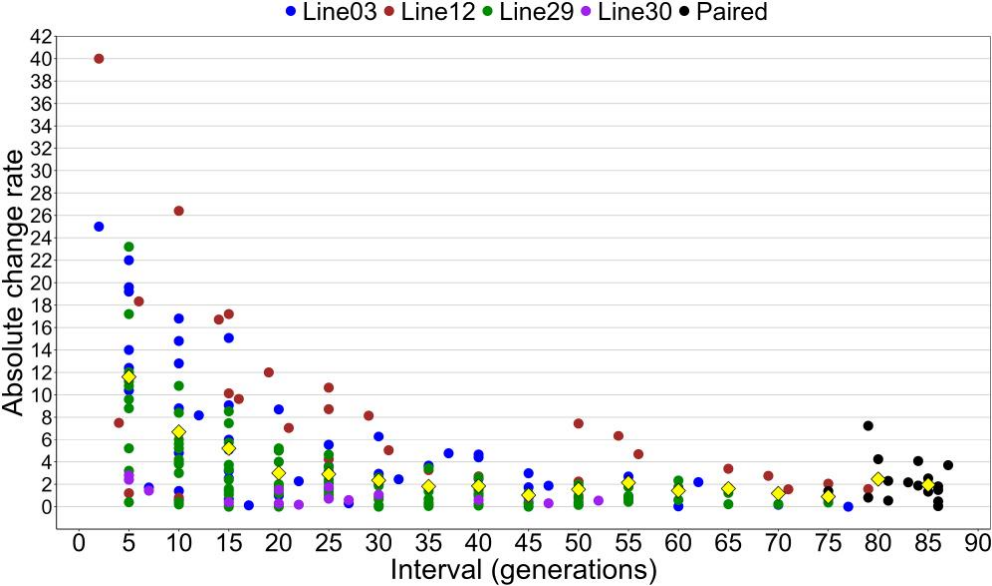
Absolute change rate per generation of diploid *28S* copy number in *D. obtusa* MA lines. Change rate is calculated as the difference in copy number divided by the difference in the number of generations (interval) between samples. Colored dots represent the 4 FG lines sampled approximately every 5 generations up to generation 95. Change rate was calculated for all pairs of samples within an FG line. Black dots represent the 16 lines sampled at generation 5 and ∼generation 87 (paired). Yellow diamonds indicate the mean absolute change rate for each interval, which includes values for intervals 2 generations above and 2 generations below the nearest 5-generation interval. For example, mean absolute change rate at interval 10 includes absolute change rates for intervals of 8 to 12 generations, while interval 15 includes absolute change rates for intervals of 13 to 17 generations.

The absolute change rate in the FG lines and paired samples varied depending on the interval measured, with shorter intervals being particularly important for capturing large changes (Fig. 3). There was a clear trend of lower absolute change rates at longer intervals, with a mean rate of about 12 copies at an interval of 5 generations, 7 copies at an interval of 10 generations, and 5 copies at an interval of 15 generations. The rate further decreased to approximately 3 copies at an interval of 20 generations and then stabilized to about 2 copies per generation after that.

### rDNA variation

A total of 58 SNPs were identified in the *D. obtusa* MA samples, and they were distributed across all 5 rDNA regions: 4 SNPs in *18S*, 6 in *28S*, 21 in IGS1, 11 in IGS2, and 16 in IGS3 (Supplementary Table 4 in Supplementary File 2, Supplementary Fig. 3 in Supplementary File 3). Two of the *18S* SNPs were in expansion helix V4 while the other 2 were in V7, based on the secondary structure of the *D. pulex 18S* gene (Crease and Colbourne 1998). One SNP in the *28S* gene was located between helices B13 and B3, 3 were in variable region V8, and 2 were in V12, based on the helix numbering system of Wuyts et al. (2001). Based on these 58 SNPs, haploSep identified 5 haplotypes (Supplementary Fig. 4a in Supplementary File 3 and Supplementary File 4) with between 16 and 30 nucleotide differences between them (mean = 25.4). Haplotypes 2 and 4 formed a clade as did haplotypes 3 and 5. If the network was rooted at the longest branch between these 2 clades, haplotype 1 would form the sister group to the haplotype 2/4 clade (Supplementary Fig. 4a in Supplementary File 3).

**Fig. 4.**
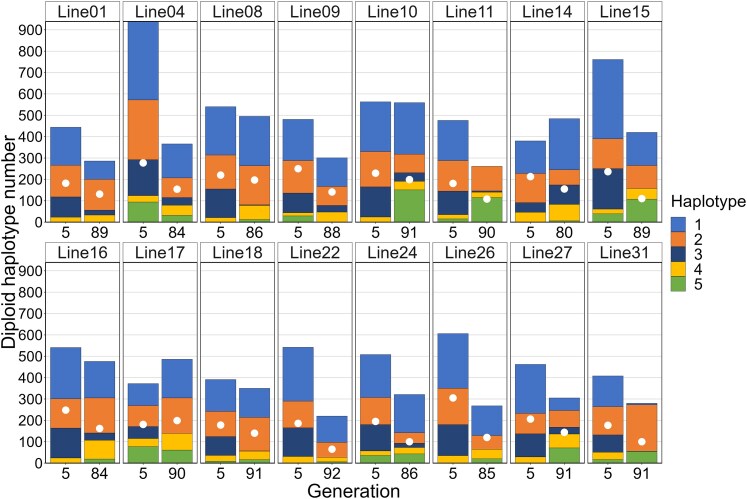
Distribution of haplotypes across 16 *D. obtusa* MA lines, each sampled at both generation 5 and generation ∼87 (paired lines). Total diploid copy number is based on the *28S* gene. The white dots represent the diploid copy number of the *Pokey* 3′ end.

Most samples contained at least 4 of the 5 haplotypes, and no single haplotype reached fixation in any of the samples. Haplotypes 1 and 2 were usually the most common in the MA05 samples (Fig. 2), while haplotypes 4 and 5 were generally at low frequencies with expected heterozygosity in these samples ranging from 0.64 to 0.78 (mean = 0.70; Supplementary Table 1 in Supplementary File 2). Haplotypes 1 and 2 remained the most common in the MA87 samples, but many showed a noticeable increase in haplotypes 4 and 5 (Fig. 2), with expected heterozygosity ranging from 0.194 to 0.787 (mean = 0.633). Haplotype frequencies in the 2 samples from line 4 were similar despite the substantial reduction in copy number at generation 84 (Fig. 4), with expected heterozygosity only decreasing slightly from 0.715 to 0.714. Similarly, copy number was high in line 15 (762) with 5 haplotypes at generation 5, but that declined to 420 copies and 4 haplotypes by generation 89. Despite the loss of haplotype 3, heterozygosity increased from 0.664 to 0.718.

**Fig. 5.**
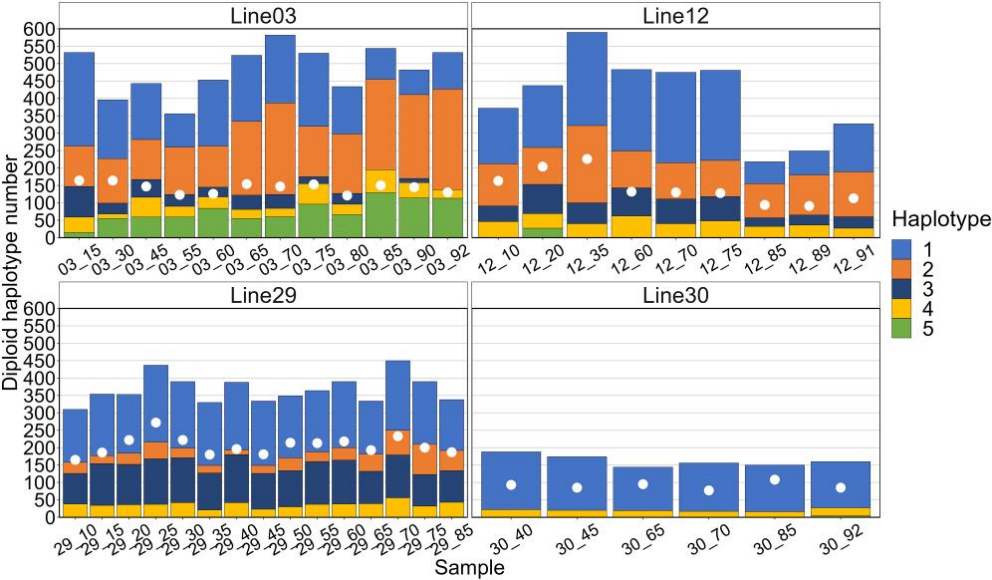
Distribution of haplotypes across samples from the 4 FG MA lines of *D. obtusa*. Total diploid copy number is based on the *28S* gene. The white dots represent the diploid copy number of the *Pokey* 3′ end.

The FG lines 3, 12, and 29 (Fig. 5) showed relatively high haplotype variation, and heterozygosity in line 12 even increased slightly from 0.632 to 0.679 when haplotype 1 and copy number declined after generation 75. Line 29 displayed a lower frequency of haplotype 2 and higher frequency of haplotype 3 compared to the other 2 lines with modest changes in heterozygosity over 75 generations (0.588 to 0.695). Compared to the other FG lines, line 30 showed substantially reduced copy number and haplotype variation at generation 40 and beyond. Haplotype 1 exceeded a frequency of 80% and haplotypes 2, 3, and 5 were lost, suggesting that this line lost most of its haplotype variation when diploid copy number decreased to <200.

Regression analysis indicated a very weak but significant relationship between expected heterozygosity and *28S* diploid copy number (*R*² = 0.225, *P* < 0.0001), with a regression slope of 0.00001 (Supplementary Fig. 5 in Supplementary File 3). However, when line 30 was excluded from the analysis, the correlation decreased considerably (*R*² = 0.037, *P* = 0.043), suggesting that line 30 had a disproportionate impact on the overall trend.

### Recombination rate

When the G-test comparing haplotype distribution between consecutive samples was significant after correcting the *P*-values, 1 recombination event was counted for the interval. Based only on the G-tests, line 3 exhibited a recombination rate of 0.176 events per generation, line 12 had a recombination rate of 0.088, line 29 had a rate of 0.043, and no recombination was detected in line 30 (Supplementary Table 5 in Supplementary File 2). The average recombination rate across all lines was 0.085, but this increased to 0.098 if line 30 was excluded. At least 1 residual value was outside the range of −2 to 2 in every comparison with a significant G-test. One additional event was counted in line 12 based on the residuals (between samples 12-10 and 12-20), which increased its recombination rate to 0.101 and the mean (excluding line 30) to 0.101.

Two additional recombination events were detected based on changes in *28S* copy number (Supplementary Table 6 in Supplementary File 2), with 1 additional event each in lines 29 and 30. After including these events, the total recombination rate based on all tests was 0.176 for line 3, 0.101 for line 12, 0.057 for line 29, and 0.010 for line 30 for an average rate of 0.094 across all 4 lines and 0.107 excluding line 30 (Supplementary Table 5 in Supplementary File 2).

### Pokey copy number and rDNA variation

The diploid copy number of the *Pokey* transposase gene in the 91 MA samples varied from 69 to 503 (mean = 171.5) while the copy number of the 3′ end varied from 65 to 537 (mean = 174.1) (Fig. 1; Supplementary Table 1 in Supplementary File 2). The copy number of the 2 regions was strongly correlated (*R*^2^ = 0.973, *P* < 0.0001), with a regression slope of 0.842 (Table 1; Supplementary Fig. 6 in Supplementary File 3) suggesting that some elements may not have complete transposase genes. Although it was not possible to differentiate *Pokey* elements inside and outside rDNA, previous work suggested that most copies in *D. obtusa* occur inside rDNA (LeRiche et al. 2014). Thus, we did a regression analysis of copy number of the *28S* gene and each *Pokey* region and found a strong positive correlation with *R*^2^ = 0.59 (*P* < 0.00001) for both regions. The regression slopes were 0.26 for the transposase gene and 0.31 for the 3′ end (Table 1; Supplementary Fig. 7 in Supplementary File 3).

**Table 1.** Regression analysis of diploid rDNA haplotype number and diploid *Pokey* 3′ end number in 90 samples from *D. obtusa* MA lines.

Predictor variable	Response variable	*R* ^2^	Slope	*P*-value
*Pokey* 3′ end	*Pokey* transposase gene	0.973	0.842	7.39E−72
Total *28S* gene	*Pokey* 3′ end	0.591	0.307	3.30E−19
Total *28S* gene	*Pokey* transposase gene	0.586	0.261	5.97E−19
rDNA haplotype 1	*Pokey* 3′ end	0.477	0.597	2.24E−14
rDNA haplotype 2	*Pokey* 3′ end	0.145	0.337	1.14E−04
rDNA haplotype 3	*Pokey* 3′ end	0.646	0.960	5.53E−22

Diploid rDNA haplotype numbers are based on the *28S* gene.

We identified 113 SNPs in the *Pokey* sequences (35 in the transposase gene and 77 in the 3′ end; Supplementary Table 4 in Supplementary File 2). HaploSep identified 6 haplotypes based on these SNPs with haplotypes 1 and 2 being most common and haplotypes 4, 5, and 6 each having a mean frequency across all samples <5% (Supplementary Table 1 in Supplementary File 2). Although rare overall, haplotypes 5 (mean number = 5.9, range = 0 to 15) and 6 (mean number = 2.5, range = 0 to 14) tended to form a larger proportion of the elements in a sample as the total *Pokey* copy number decreased and persisted even after the large decreases in rDNA copy number in line 30 (Supplementary Figs. 8 to 10 in Supplementary File 3). This suggests that these *Pokey* haplotypes tend to occur in low-recombination regions of the rDNA array. Expected heterozygosity based on *Pokey* haplotype frequencies ranged from 0.49 to 0.75 (mean = 0.63), which is slightly lower than values based on rDNA haplotypes.

The average number of differences between haplotypes based on the 113 SNPs was 52.7 with a range of 34 to 72 (Supplementary File 4). The haplotypes formed 3 clades on a TCS network (Supplementary Fig. 4b in Supplementary File 3): one containing haplotypes 1 and 4, a second containing haplotypes 2 and 3, and a third containing 2 of the 3 low-frequency haplotypes, 5 and 6 (Supplementary Table 1 in Supplementary File 2).

To determine if *Pokey* elements were associated with a particular rDNA haplotype, we performed a regression analysis between each of the 3 most common haplotypes (1, 2, 3) and the *Pokey* 3′ end. The results were all significant with the highest slope associated with haplotype 3 followed by haplotype 1 (Table 1). Indeed, most, if not all, of the *Pokey* elements in the samples from line 30 must be inserted in haplotype 1 as haplotypes 2 and 3 were lost after the substantial decrease in total copy number before generation 40 (Fig. 5). However, there are samples in which the number of *Pokey* sequences substantially exceeds the combined number of haplotypes 1 and 3 (e.g. 11-90, 31-91; Fig. 4), suggesting that elements inserted in haplotype 2 account for these. Overall, these results suggest that there were a substantial number of *Pokey* inserts in all 3 of the most common rDNA haplotypes such that *Pokey* persisted in an MA line regardless of which haplotype decreased in frequency over time.

The proportion of *28S* genes with *Pokey* inserts based on the 3′ end ranged from 24.4% to 72.0% with a mean = 42.5% and median = 42.2% (Supplementary Table 1 in Supplementary File 2). The diploid *28S* copy number of the sample with the highest proportion of *Pokey* elements (30-85) was only 150, which would leave <50 functional *28S* genes assuming all the elements were located in rDNA. Indeed, the diploid *28S* copy number of all samples from line 30 ranged from 144 to 188 with *Pokey* 3′ end copy numbers from 77 to 108 (49% to 72%; Fig. 5; Supplementary Table 1 in Supplementary File 2).

## Discussion

### Copy number variation

We observed substantial variation in the diploid number of rDNA regions in samples from *D. obtusa* MA lines propagated apomictically for fewer than 100 generations. This variation underscores the dynamic nature of rDNA, with a diploid change rate as high as 150 copies per generation in 1 sample (assuming an initial copy number of 512). Despite such large increases early in the MA experiment, we found that rDNA copy number tended to decrease overall with the most substantial change in line 30, which decreased to 188 by generation 40 and remained consistently low thereafter. Moreover, only 4 of the MA87 samples (3 of which were from line 3) exceeded 500 copies by generation 92 with a maximum of 560.

The change rate of diploid *28S* copy numbers varied across generations in the FG lines, suggesting no strong directional bias overall. Of the 38 estimates between consecutive samples, 20 were positive, so every line experienced both increases and decreases in rDNA copy number with no apparent bias over time. However, estimates from 14 of the 16 paired lines were negative, which is consistent with the overall trend toward decreased copy number in the MA87 samples. Even when rDNA copy did increase within an FG line, the capacity for increase was limited. This contrasts with the substantial increase observed in 3 lines (4, 15, 21) after only 5 generations suggesting that the capacity for rDNA expansion did exist in the progenitor female. However, no increases of that magnitude were observed across 5-generation intervals from later sampling times suggesting that the capacity for copy number expansion had declined over time. These results differ from those obtained for 2 lineages of *D. pulex* MA lines cultured for up to 200 generations (Elguweidi et al. 2025) in which the estimated copy number of the progenitor females (299 and 378) was lower than the *D. obtusa* progenitor (512) and fell at the low end of the diploid *28S* range (346 to 4,904) in *D. pulex* natural populations (Elguweidi and Crease 2024). Indeed, the *D. pulex* sample with the highest *28S* number (1,268) in the MA lines was taken at generation 115 (Elguweidi et al. 2025).

Sharp et al. (2023) also observed a general trend of rDNA copy number decline over ∼1600 generations in 220 MA lines of *S. cerevisiae*. They found mutations in genes that are not directly involved in rDNA maintenance (e.g. *RDH54*, *RIF1*, and *MUS81*) but are critical for repairing DNA DSBs and facilitating homologous recombination. They argued that mutations in such genes could affect rDNA copy number regulation. Notably, the *S. cerevisiae* MA line with the highest copy number carried a mutation in the *DPB4* gene, which is linked to DNA replication and repair, and potentially affects regulation of rDNA amplification. However, such cases appeared to be rare and were not directly tested in the study. Even so, this underscores how genetic variation in genes outside rDNA could indirectly impact rDNA stability. A comparison of mutations in the genomes of *D. obtusa* MA lines sampled more than once could explore the possibility that mutations in genes that control DNA repair and recombination are associated with the decline in rDNA copy number we observed over time.

The copy number variation we observed in the *D. obtusa* MA lines is similar to that observed by LeRiche et al. (2014). For example, they reported that diploid *28S* copy number in line 3 ranged from 327 to 586 while our estimates ranged from 368 to 600. Similarly, they reported a range of 211 to 505 for line 12 compared to our estimates of 226 to 602, and a range of 367 to 462 for line 29 compared to our estimates of 310 to 450. In addition, LeRiche et al. analyzed 21 samples at generation ∼87 and obtained a range of 147 to 597 (mean = 408) compared to our 36 samples with a range of 150 to 560 (mean = 372). Thus, estimates based on the 2 methods are similar even though the qPCR copy number denominator was based on amplification of ∼20 bp of only 2 SCGs, compared to sequencing read depth across 1 exon from each of 16 SCGs. Despite the similar range of values in the 2 studies, *R*^2^ values between sequencing and qPCR estimates for the same samples were only 0.674 for *18S* and 0.641 for *28S*. Because the correlation between genes within samples was higher with sequencing (*R*^2^ = 0.996) than qPCR (*R*^2^ = 0.942) (Supplementary Fig. 11 in Supplementary File 3), it may be tempting to suggest that sequencing provides more accurate estimates of rDNA copy number than qPCR. However, we did see variation in read depth across the 16 exons (Supplementary Fig. 12 in Supplementary File 3), which could reduce the accuracy of copy number estimates based on short-read sequences. The precision of these estimates could be tested by employing different sets of exons.

Rabanal et al. (2017) compared haploid *18S* copy number estimates obtained by qPCR with those from short-read sequencing data in 10 samples of *Arabidopsis thaliana* MA lines and found a higher correlation between methods (*R*² = 0.88) than we found. They argued that the strong correlation between methods they observed supports the reliability of qPCR estimates in capturing rRNA gene copy number variation, and our results support this. Based on studies of yeast and humans, Hall et al. (2022) argued that digital droplet PCR (ddPCR) provides improved accuracy, resolution, and replicability compared to qPCR. However, Sharp et al. (2023) used both short-read sequencing and ddPCR to estimate rDNA copy number in 10 samples from *S. cerevisiae* MA lines and reported a correlation between methods of 0.78, which is about midway between the values obtained by us and Rabanal et al. (2017). There are sources of error associated with all 3 methods, but overall, the strong correlation between sequencing and PCR methods suggests that all of them can provide reasonable estimates of rDNA copy number.

In our analysis of *D. pulex* MA lines (Elguweidi et al. 2025), the shortest interval between samples (30 generations) showed a high mean absolute change rate (∼8/generation), with a broad range of values (0 to 15). In contrast, we observed reduced variation in absolute copy number change rates at long intervals (∼77 and 107 generations). Similarly, Rabanal et al. (2017) found that rDNA copy number is highly unstable in MA lines of *A. thaliana*, with a range of diploid *28S* copy number from 576 to 1523 across ten lines after 30 generations. This high level of rDNA copy number instability in *A. thaliana* aligns with our observations of change rate over short intervals in *Daphnia.* Together, these findings highlight the importance of measuring change rates over as short an interval as possible if the goal is to determine the magnitude and frequency of recombination events.

If there is little bias in the direction of copy number change, change rates measured over long intervals will be very low and the mean across lines will approach 0 as increases and decreases tend to cancel each other out over time. For example, the *28S* copy number of sample 29-10 was 310 and the copy number of sample 29-25 was 438, which gives an interval of 15 generations and a change rate of 12.3 copies per generation. The copy number of sample 29-70 (450) is similar to that of 29-25, but the interval between these samples is much longer at 60 generations. This gives a substantially lower change rate of 2.3 copies per generation because the changes (both increases and decreases) that occurred between samples 29-25 and 29-70 are obscured. Indeed, the absolute change rate between the first and last samples of all 4 FG lines was <1 copy per generation despite substantial changes in both directions between intermediate samples.

Patterns of copy number variation in the spacers (IGS1, IGS2, and IGS3) of the *D. obtusa* MA lines followed those observed in the *18S* and *28S* genes, as expected if each rDNA repeat contains 1 copy of each region. However, the correlation between copy number estimates of the rRNA genes and the IGS regions and between IGS regions was lower than that between the 2 rRNA genes. A similar pattern was observed in both natural populations and MA lines of *D. pulex* (Elguweidi and Crease 2024; Elguweidi et al. 2025). The copy number differences between the genes and the IGS regions may be due to experimental artifacts, such as differences in read mapping efficiency that affect read depth. For example, we observed reduced average read depth across IGS1 in one of the *D. pulex* MA lineages we studied (Elguweidi et al. 2025) due to a 140 bp deletion relative to the reference sequence. On the other hand, average read depth could be overestimated if some IGS sequences occur at chromosomal locations outside rDNA (De Lucchini et al. 1988), although this phenomenon was not observed in the first sequenced *D. pulex* genome (Colbourne et al. 2011).

### rDNA variation

Our analysis of 58 SNP frequencies across *D. obtusa* MA lines identified 5 haplotypes. As expected, the SNPs were primarily located in the spacer regions and the expansion segments and variable regions of the rRNA genes. These regions are thought to be under less functional constraint than the core regions of the *18S* and *28S* genes (Gerbi 1986), and thus, the variation is likely to be neutral or nearly neutral. This is consistent with patterns observed in *D. pulex* natural populations (Elguweidi and Crease 2024) and MA lines (Elguweidi et al. 2025), as well as other organisms (e.g. Stage and Eickbush 2007). Since all the MA lines were derived from a single progenitor female, we expected most of them to display the 5 haplotypes we identified. Nevertheless, variation in haplotype distribution was observed with contraction and expansion of different haplotypes among lines. Given that selection is reduced in MA lines, these changes are likely the result of random sampling of variant rDNA arrays created by unequal recombination during apomixis.

Ganley and Kobayashi (2011) found that rDNA units are frequently gained or lost, sometimes as often as every cell division. When a repeat unit duplicates, it typically remains close to the original but can occasionally spread further along the array such that new rDNA variants can be distributed in a combination of small clusters and dispersed copies. The former pattern likely explains the expansion of low-frequency haplotype 5 in some *D. obtusa* MA lines (e.g. 3, 11, 25, 47). On the other hand, large deletions that remove substantial portions of an rDNA array create a bottleneck effect by reducing the variety of repeat units, which accelerates the spread of remaining types and leads to greater uniformity among rDNA repeats. This is undoubtedly what occurred in line 30, which lost all but 2 haplotypes, including 2 of the 3 most common ones.

There is a slight positive and significant association between *28S* copy number and expected heterozygosity in our data. Only about 3.7% of the variation in heterozygosity is explained by copy number, suggesting that it is not a strong predictor of heterozygosity. For example, we observed cases where copy number decreased with little effect on haplotype frequency or overall heterozygosity (e.g. line 4) while in other lines, decreased copy number resulted in loss of haplotypes and reduced heterozygosity (e.g. lines 30, 31). This weak association suggests that the common haplotypes tend to occur in multiple clusters that are dispersed along the rDNA array. This contrasts with the situation in *D. pulex* MA lines where clustering of haplotypes appeared to be more pronounced as changes in copy number often caused substantial changes in the distribution of haplotypes within individuals (Elguweidi et al. 2025).

**Fig. 6.**
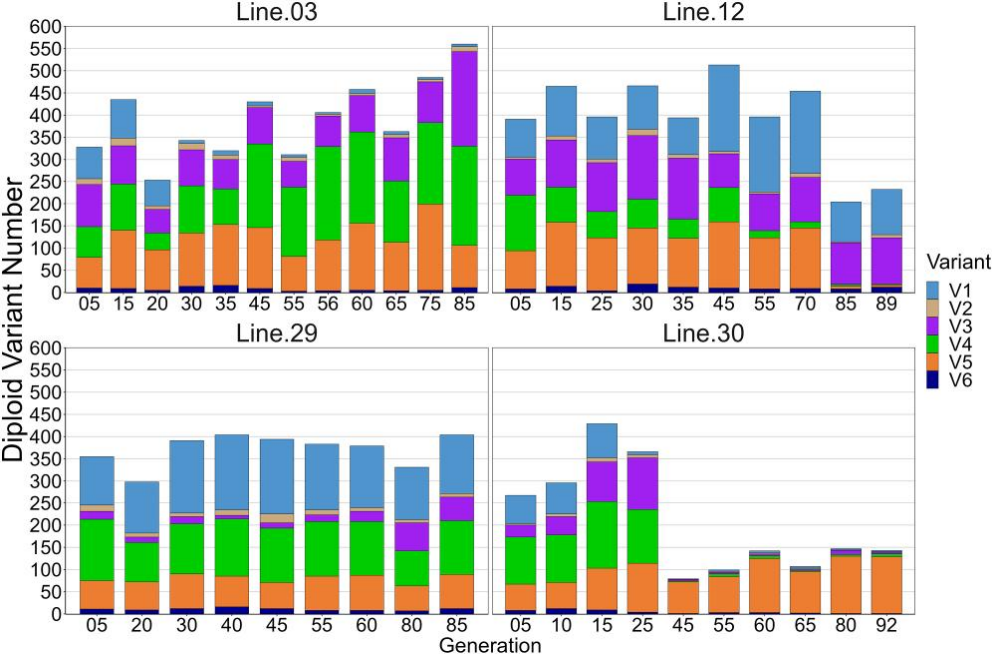
Changes in the frequency of length variants in an expansion segment of the *18S* gene in the 4 FG MA lines of *D. obtusa.* Length variant frequency data from McTaggart et al. (2007) was combined with rDNA copy number data (obtained using qPCR) from McTaggart et al. (2007) and LeRiche et al. (2014) for a total of 41 samples. The average copy number was used for the 11 samples that were analyzed by both groups. Differences between these estimates ranged from 1 to 51 (mean = 16). In all but 2 cases, the McTaggart et al. values were smaller than those reported by LeRiche et al.

McTaggart et al. (2007) estimated the frequency of short indels in expansion segment 43/e4 of the *18S* gene and reported the presence of 6 length variants that varied in frequency across lines and within the FG lines. They noted that the most significant change in diploid rDNA copy number occurred between generations 25 and 60 in line 30, with the diploid *18S* copy number decreasing from 380 to 140 and remaining low thereafter. LeRiche et al. (2014) also observed the substantial drop in *18S* copy number after generation 25 but found it had occurred by generation 45. We found that this drop occurred even earlier, sometime between generation 25 and 40. This decline was accompanied by an increase in haplotype 1 frequency to over 80%, which is comparable to the observed increase in *18S* V5 frequency to 90% reported by McTaggart et al. (2007). We used the data from McTaggart et al. and LeRiche et al. to create barplots of copy number (estimated using qPCR) and variant frequency in the FG lines (Fig. 6) and found that the pattern of variant frequency changes closely mirrors the pattern of haplotype frequency changes we observed, even though the indel variants occurred in a small region of the *18S* gene and the haplotypes we identified were based on SNP variation across most of the rDNA repeat.

### Recombination rate

We followed the same method as McTaggart et al. (2007) to estimate rDNA recombination rate, and found an overall mean rate of 0.098 excluding line 30 and a mean of 0.085 including line 30. Mutations accumulating over time may have contributed to variation in the recombination rate between lines as each one evolved independently. For instance, line 3 showed the highest recombination rate of 0.176 events per generation, while line 30 lacked detectable recombination events. However, all line 30 samples were taken after the substantial drop in copy number and near fixation of haplotype 1 between generations 25 and 40, which would make it difficult to detect recombination events even if they did occur.

Based on a diploid copy number of 400, McTaggart et al. (2007) found a mean recombination rate of 0.074 based on lines 3, 12, and 29. They also detected recombination in line 30 based on samples taken before the substantial drop in copy number giving an average across all 4 lines of 0.068. Overall, these results are similar to ours even though they are based on indel variants in a small region of the *18S* gene.

McTaggart et al. (2007) suggested that using G-test and residual methods to estimate rDNA recombination rate is conservative as only 1 event is counted per interval. This avoids inflating the rate when a single event alters the frequency of multiple haplotypes. On the other hand, multiple events could occur within an interval and would not be counted. Moreover, recombination events such as equal exchanges between sister chromatids or those that do not significantly alter haplotype frequencies would not be detected at all. Similarly, if a crossover occurs between chromatids from homologous chromosomes and both parental or both recombinant chromatids segregate into the same daughter cell, the event would not be detected because the relative frequency of variants and the total copy number would not change (McTaggart et al. 2007). Thus, it is very likely that we underestimated the rDNA recombination rate in the *D. obtusa* MA lines.

Recombination rates in rDNA have only been estimated in a small number of species. Sharp et al. (2018) estimated an rDNA recombination rate of approximately 0.0023 events per generation in MA lines of *S. cerevisiae*, while earlier studies reported rates up to 0.001 (Szostak and Wu 1980), 0.0013 (Merker and Klein 2002), and 7.4 × 10^−5^ to 7.5 × 10^−^  ^4^ (Kobayashi et al. 2004). Recombination rate in *Drosophila* rDNA has been estimated at about 0.0001 events per generation (Williams et al. 1989). Including our estimates, this range varies by 4 orders of magnitude and underscores the species-specific nature of rDNA recombination rate. On the other hand, differences in measurement approaches, such as the number of generations between samples and detection sensitivity, may also have contributed to the wide range of reported rates. Future studies could benefit from more standardized methodologies to ensure more accurate estimates of rDNA recombination rate, although this may be difficult to achieve with all organisms.

### Pokey copy number and sequence variation

The transcription domain model of *R2* retrotransposon regulation created by Zhang et al. (2008) and expanded by Zhou et al. (2013) was used to explain the fact that the retrotransposons *R1* and *R2* have persisted and remained active in the *28S* genes of *Drosophila* and other arthropods for millions of years even though inserted genes are not functional. The model suggests that the location of transcriptionally active rDNA repeats (the transcription domain) is based on the longest block of continuous uninserted rDNA repeats. The remaining repeats are silenced by the formation of heterochromatin. If R-elements are clustered, they will tend to occur in heterochromatinized regions and will not be expressed, in which case they cannot transpose. Moreover, since the recombination events that change rDNA copy number generally occur in transcriptionally active regions (Averbeck and Eickbush 2005; Zhou et al. 2013), the R-elements will not usually be involved in these events and their numbers will tend to be stable. However, if the R-elements are dispersed so that the longest block of uninserted rDNA repeats is shorter than a transcription domain, then some active repeats will contain R-elements, which can be transcribed and whose copy number is more likely to be affected by the recombination events that change rDNA copy number.

In Lepidoptera, *R1* and *R2* also insert into rDNA arrays, but their distribution is more variable than in *Drosophila*. Long-read sequencing shows that these elements are present in some species (e.g. *Lymantria dispar*, *Bombyx mori*), but absent in others (e.g. *Spodoptera frugiperda*, *Plutella xylostella*), suggesting rapid turnover and incomplete persistence in rDNA loci (Dalíková et al. 2023). Recent work by Nelson et al. (2023) suggests this very dispersion dynamic may allow *R2* elements to transition from genomic parasites to mutualists. Their data demonstrate that *R2* insertions can stabilize rDNA arrays by serving as recombination templates that restore copy number after severe depletion.

LeRiche et al. (2014) argued that the transcription domain model also explains the long-term persistence of *Pokey* elements in *Daphnia* and the pattern of copy number variation in the *D. obtusa* MA lines even though *Pokey* is a DNA transposon and occurs outside rDNA in some *Daphnia* species (Eagle and Crease 2016). They found that the proportion of *28S* genes with *Pokey* inserts in their MA87 samples from the *D. obtusa* MA lines averaged 30%, and the copy number of *Pokey* and *28S* genes was strongly correlated (*R*^2^ = 0.52, *P* = 0.00036). We found that the proportion of inserted *28S* genes was even higher (mean = 42% across all samples) and was also significantly correlated with *28S* number (*R*^2^ = 0.59, *P* < 0.00001). This level of transposon insertion was also observed in 16 MA lines of *D. melanogaster* in which 34%, on average, of the *28S* genes contained an *R1* insert. Moreover, *R1* insert number was significantly correlated with rDNA copy number, which ranged from 140 to 310 after 400 generations (Averbeck and Eickbush 2005). Conversely, only 12% of the *28S* genes contained an *R2* element and their number was relatively stable across lines.

Based on the transcription domain model, LeRiche et al. suggested that *Pokey* elements are likely to be somewhat dispersed throughout the *D. obtusa* rDNA array, which is supported by the results of a fluorescent in situ hybridization experiment involving an individual of *D. pulex* from a population with an unusually high number of rDNA *Pokey* inserts (Figure S11 in Colbourne et al. 2011). This conclusion is also consistent with the relative stability of *Pokey* haplotype frequencies we observed in the FG lines. Overall, the strong correlation between *Pokey* number and each of the 3 most common rDNA haplotypes, along with the relative stability of haplotype frequencies despite copy number changes suggests that rDNA haplotypes in the *D. obtusa* MA lines occur in dispersed clusters that each contain *Pokey* inserts. If *Pokey* is indeed dispersed in *D. obtusa* genomes such that inserts occur in transcriptionally active rDNA repeats, the domain model predicts that we should find a negative correlation between the number of uninserted genes and the number of rRNA transcripts containing *Pokey* sequence.

The 6 samples from *D. obtusa* line 30 had low rDNA copy number (mean = 162) and a high proportion of *Pokey* inserts (mean = 57%) suggesting that they had only ∼70 functional *28S* genes, on average. Since the lowest estimate of uninserted repeats (42) occurred at generation 85 and the line was still alive at generation 92, it appears this number of functional *28S* genes was sufficient to meet the requirement for rRNA. This is consistent with studies in *Drosophila* in which it has been estimated that only 35 to 50 functional rDNA repeats are sufficient for normal development (Ye and Eickbush 2006).

## Conclusions

rDNA copy number generally declined after ∼87 generations in *D. obtusa* MA lines, with only 2 lines (4 samples) ending up above the initial value. On the other hand, there were very large increases in 3 lines within the first 5 generations. This trend underscores the importance of studying rDNA copy number variation across short intervals, as large changes might be overlooked with longer sampling intervals. The pattern of shifts in haplotype distribution suggests that the common haplotypes tend to occur in multiple clusters that are dispersed along the rDNA array. Moreover, most, if not all, of the haplotype clusters contain *Pokey* transposon inserts such that *Pokey* and haplotype copy numbers are strongly correlated. rDNA recombination rates were found to vary between 4 MA lines, with an average value of 0.09 across lines. Even though this is higher than most estimates reported for other organisms, it is also likely to be an underestimate. Future studies could analyze mutations in the genomes of the MA lines to identify associations between rDNA stability and variation at genes that influence recombination. Other studies could focus on the underlying mechanisms driving rDNA copy number fluctuations and their phenotypic effects. Overall, this work underscores the complexity of rDNA evolution and the importance of considering both short- and long-term dynamics when studying genetic variation.

## Supplementary Material

jkaf279_Supplementary_Data

## Data Availability

Genome sequence data can be found in the GenBank BioProject Database at https://www.ncbi.nlm.nih.gov/genbank/, with the corresponding accession numbers listed in Supplementary Table 1 in Supplementary File 2. The scripts and codes used for our analyses are available on GitHub at https://github.com/elguweidi/Daphnia.pulex.natural.population.git. Supplemental material available at G3 online.
