# Supplementary material for: Copy number and sequence variation in ribosomal DNA and the transposon, Pokey, in mutation accumulation lines of Daphnia obtusa
Source: G3 (Bethesda). 2025 Nov 20;16(2):jkaf279. doi: 10.1093/g3journal/jkaf279 (PMC12869080; doi:10.1093/g3journal/jkaf279)
Supplement: jkaf279_Supplementary_Data [file jkaf279_supplementary_data.zip › Supplemental_File_S3_G3-2025-406347.pdf]

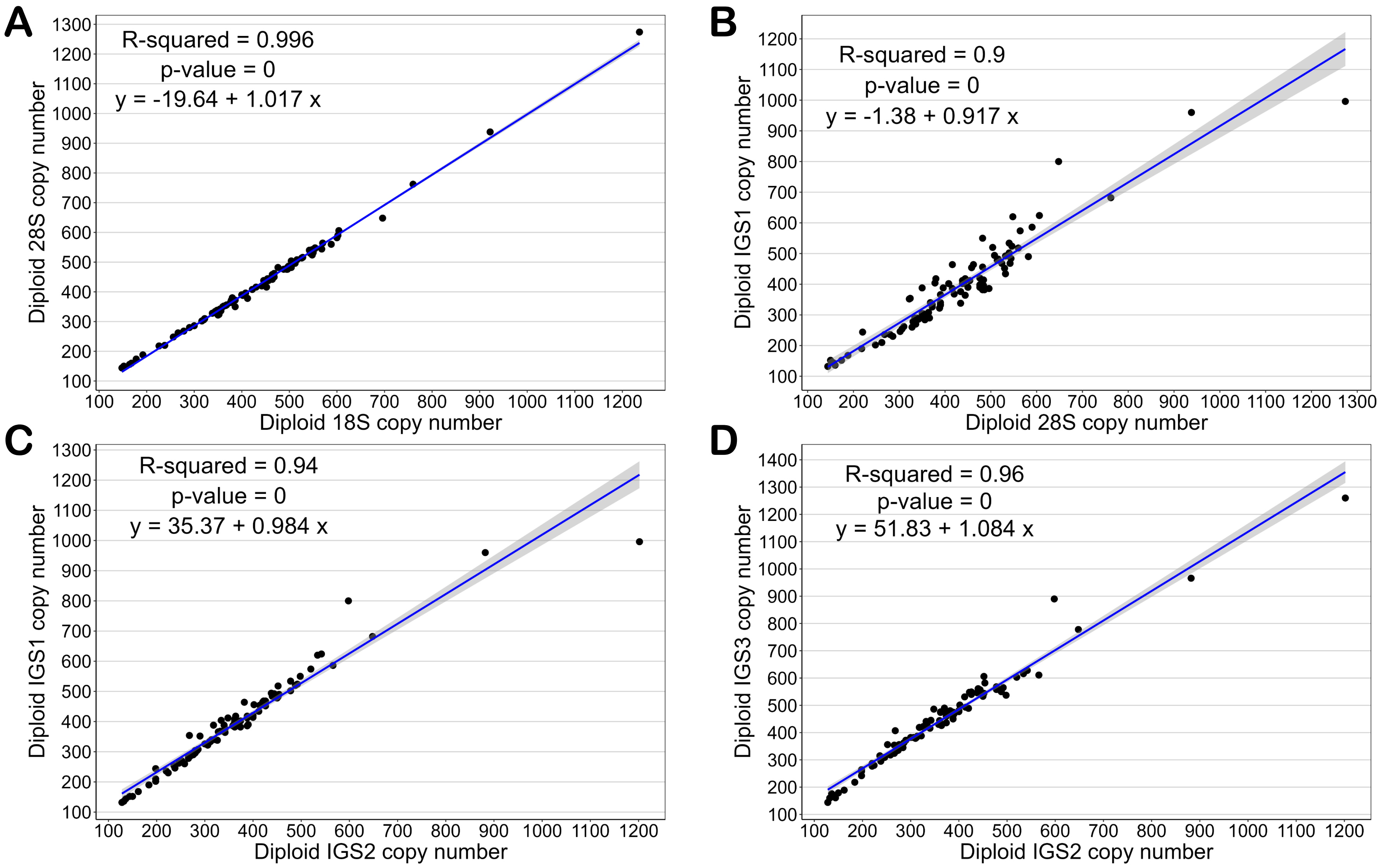

Figure S1. Regression of diploid copy number of rDNA regions in 90 samples from *D.obtusa* MA lines. The expected slope of the line is 1 if there is one copy of each region per rDNA repeat unit. **A.** 18S and 28S copy number. **B.** 28S and IGS1 copy number. **C.** IGS2 and IGS1 copy number. **D.** IGS2 and IGS3 copy number.

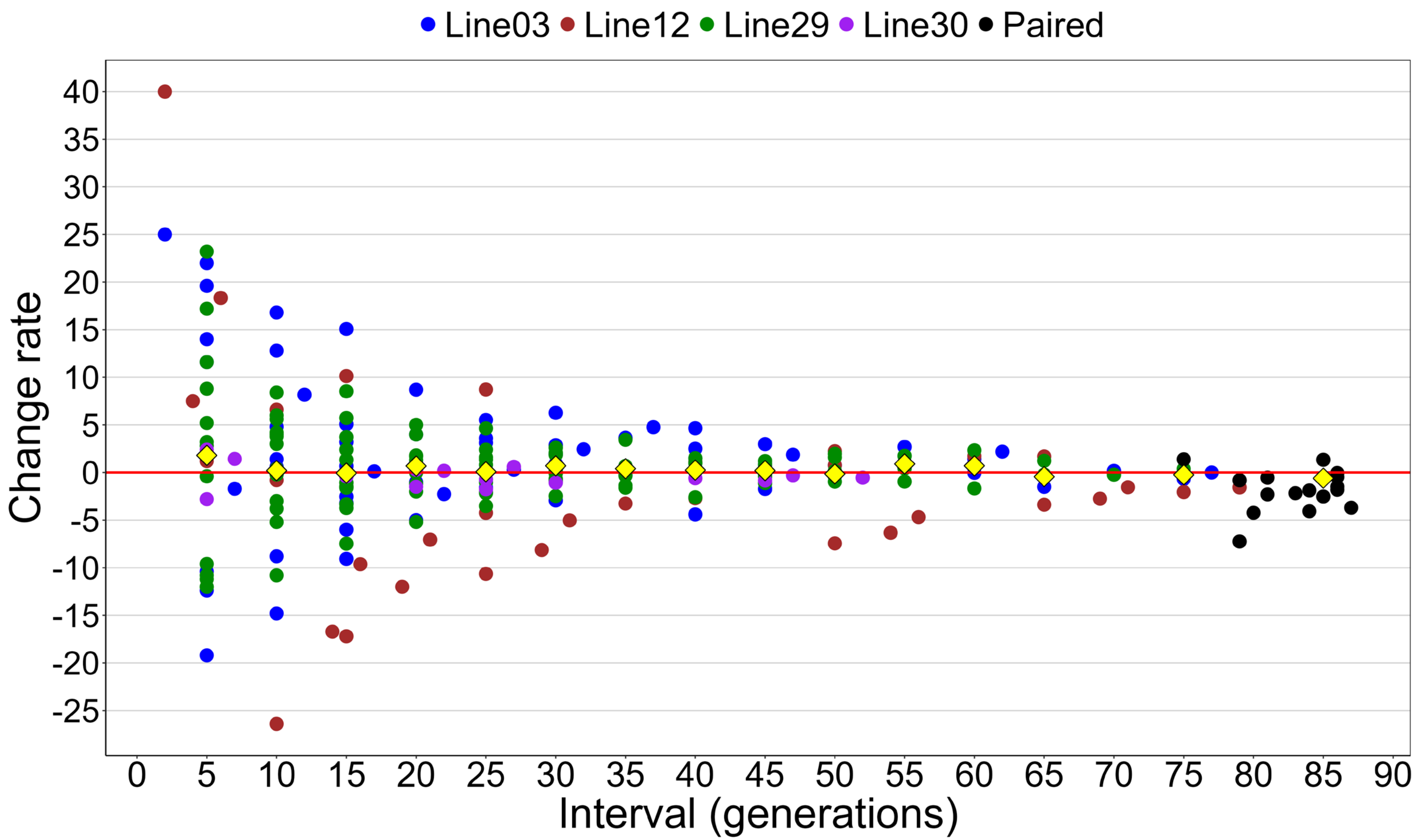

Figure S2. Change rate per generation of diploid 28S copy number in 90 samples from *Daphnia obtusa* MA lines. Change rate is calculated as the difference in copy number divided by the difference in the number of generations (Interval) between samples. Colored dots represent the four Fine grained (FG) lines sampled approximately every five generations up to generation 95. Change rate was calculated for all pairs of samples within an FG line. Black dots represent the 16 lines sampled at generation 5 and ~generation 87 (Paired). Yellow diamonds indicate the mean absolute change rate for each interval, which includes values for intervals 2 generations above and 2 generations below the nearest 5-generation interval. For example, mean absolute change rate at interval 10 includes absolute change rates for intervals of 8 to 12 generations, while interval 15 includes absolute change rates for intervals of 13 to 17 generations.

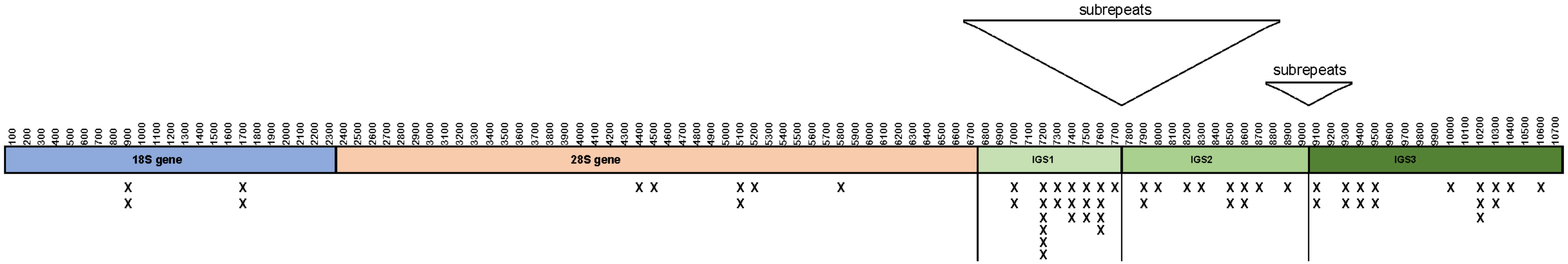

Figure S3. Distribution of 58 single nucleotide polymorphisms in the rDNA repeat of 90 samples from *Daphnia obtusa* MA lines. The rDNA repeat unit is divided into 100 nt windows. The subrepeat regions between the intergenic spacers (IGS1, IGS2 and IGS3) were omitted from analysis. The region between IGS1 and IGS2 is composed of a variable number of 3 subrepeat types; A (195 nt), B (27 nt), C (193-201 nt) (Ambrose and Crease 2011). The region between IGS2 and IGS3 is composed of a variable number of D subrepeats (71-75 nt).

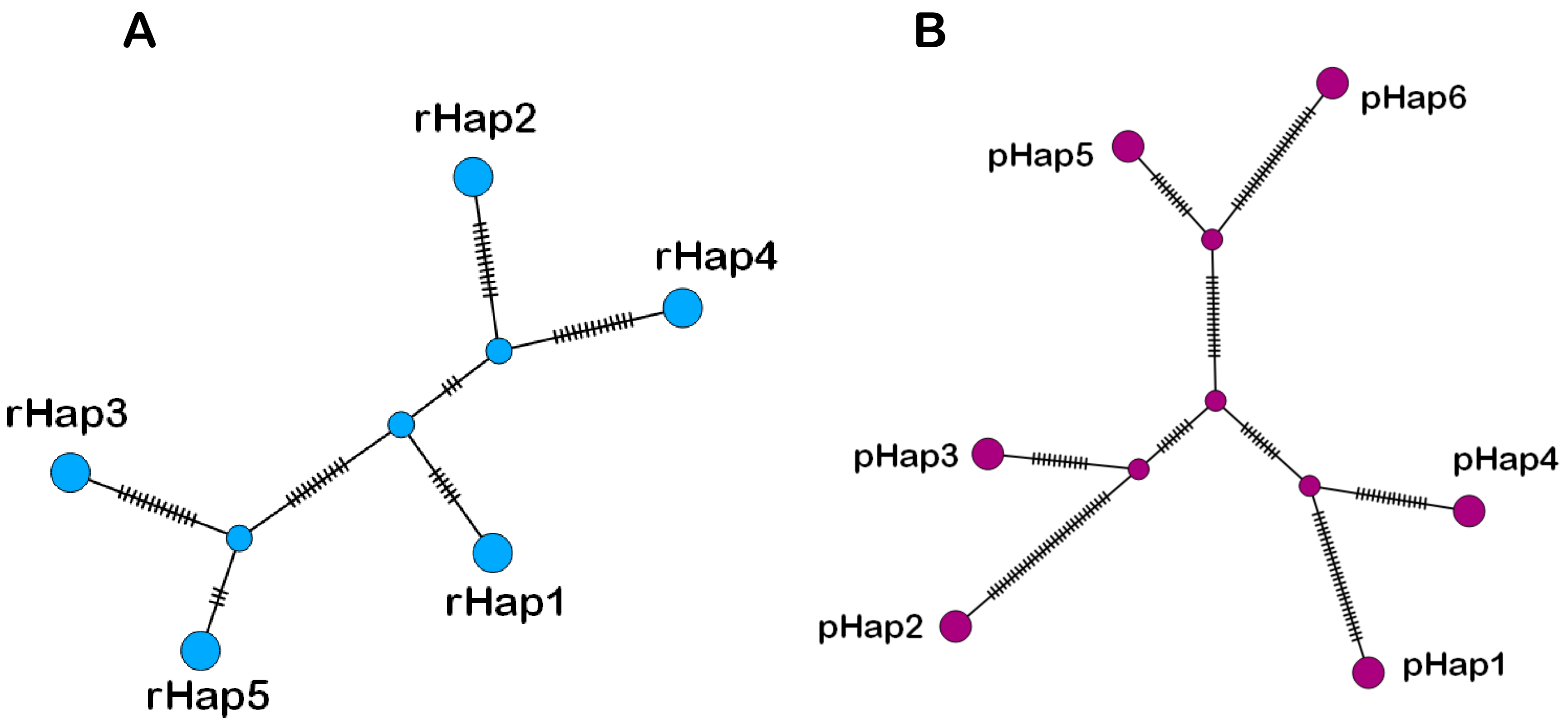

Figure S4. Haplotype networks based on rDNA and Pokey SNPs in 90 samples from *Daphnia obtusa* MA lines. **A.** Network of rDNA haplotypes based on 58 SNPs. **B.** Network of *Pokey* haplotypes based on 113 SNPs in the transposase gene and the 3′ end.

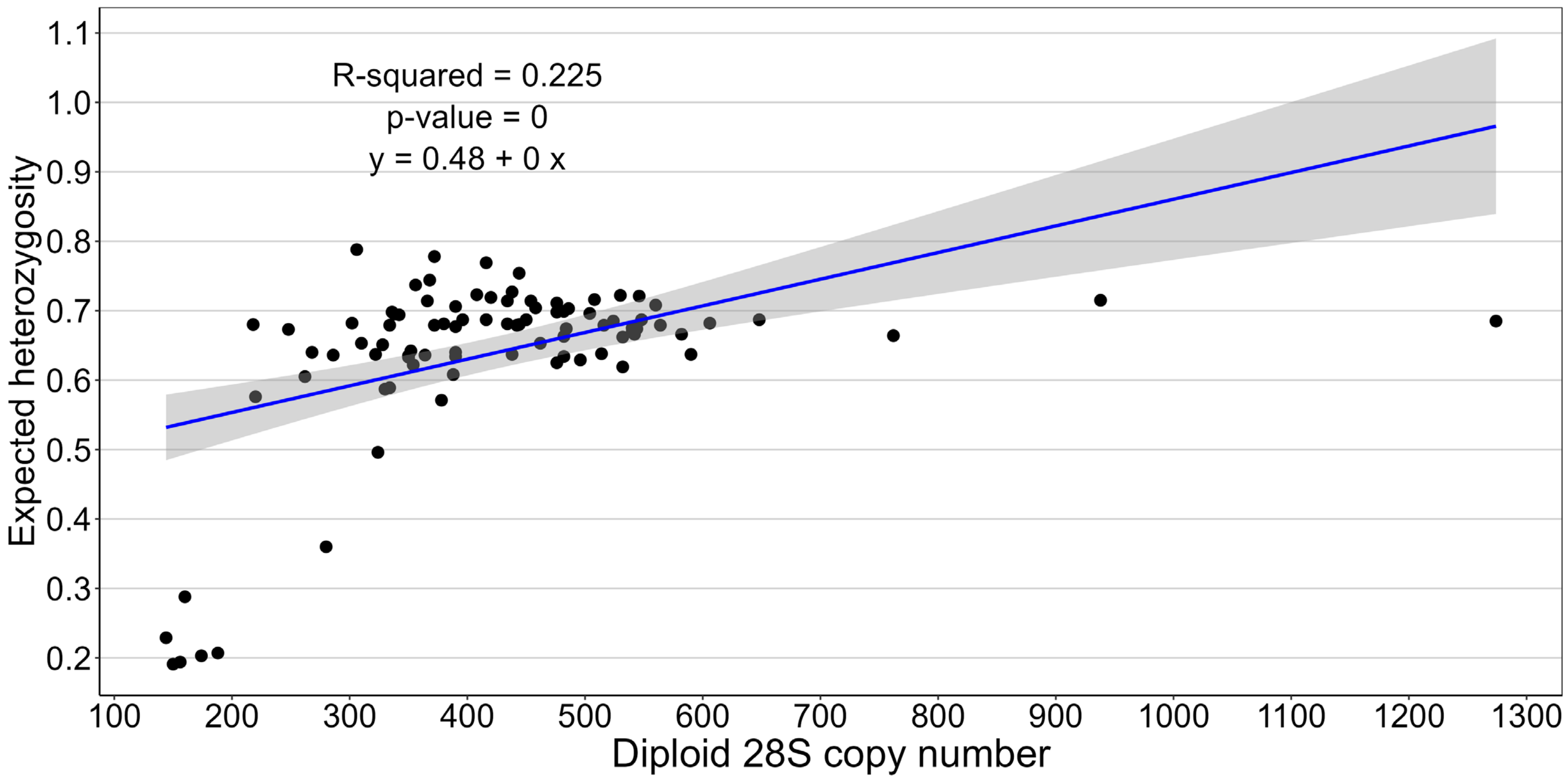

Figure S5. Regression analysis of the relationship between diploid 28S copy number and expected heterozygosity in 90 samples from *Daphnia obtusa* MA lines.

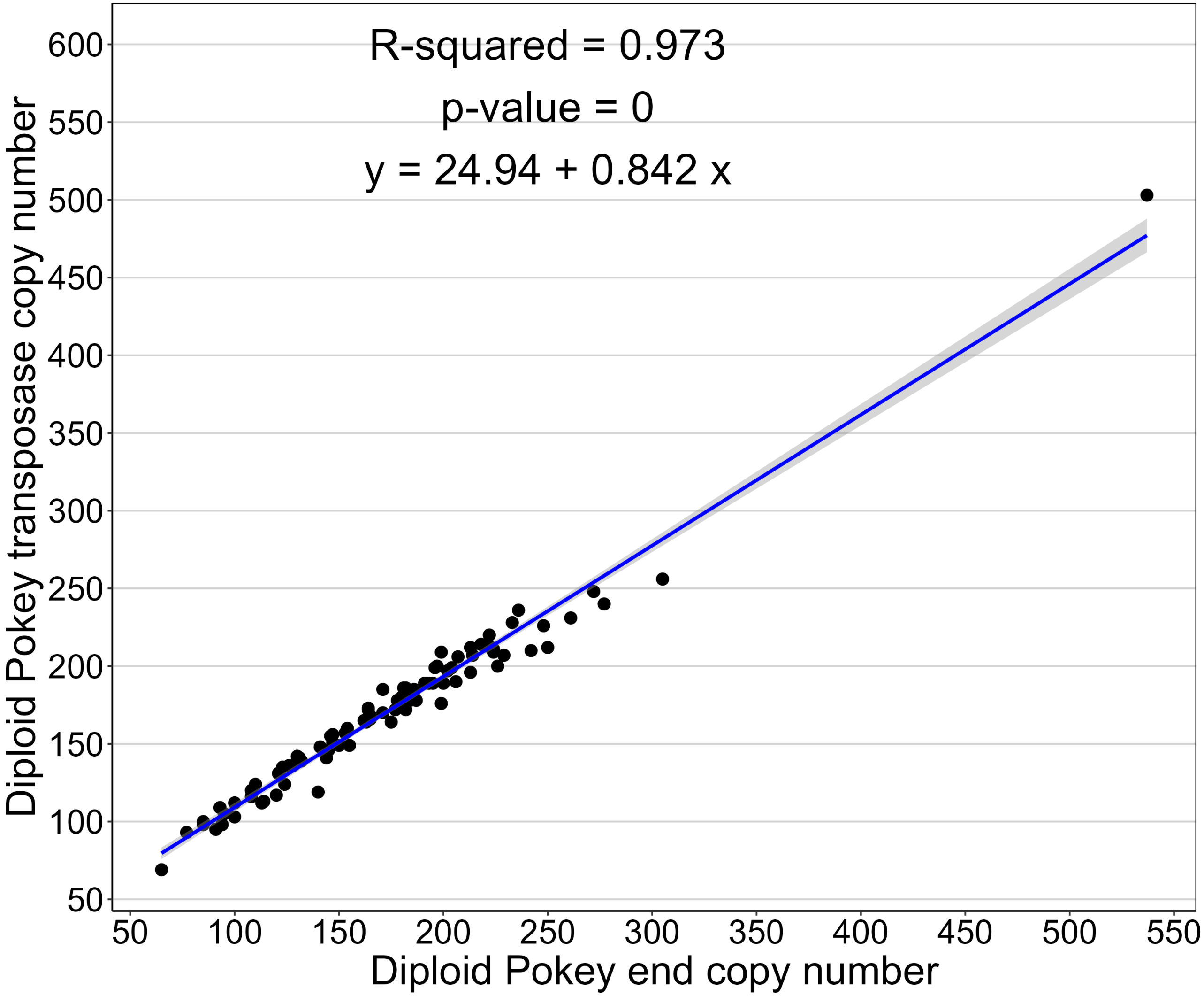

Figure S6. Regression analysis of the relationship between diploid *Pokey* 3′ end copy number and diploid *Pokey* transposase gene copy number in 90 samples from *Daphnia obtusa* MA lines.

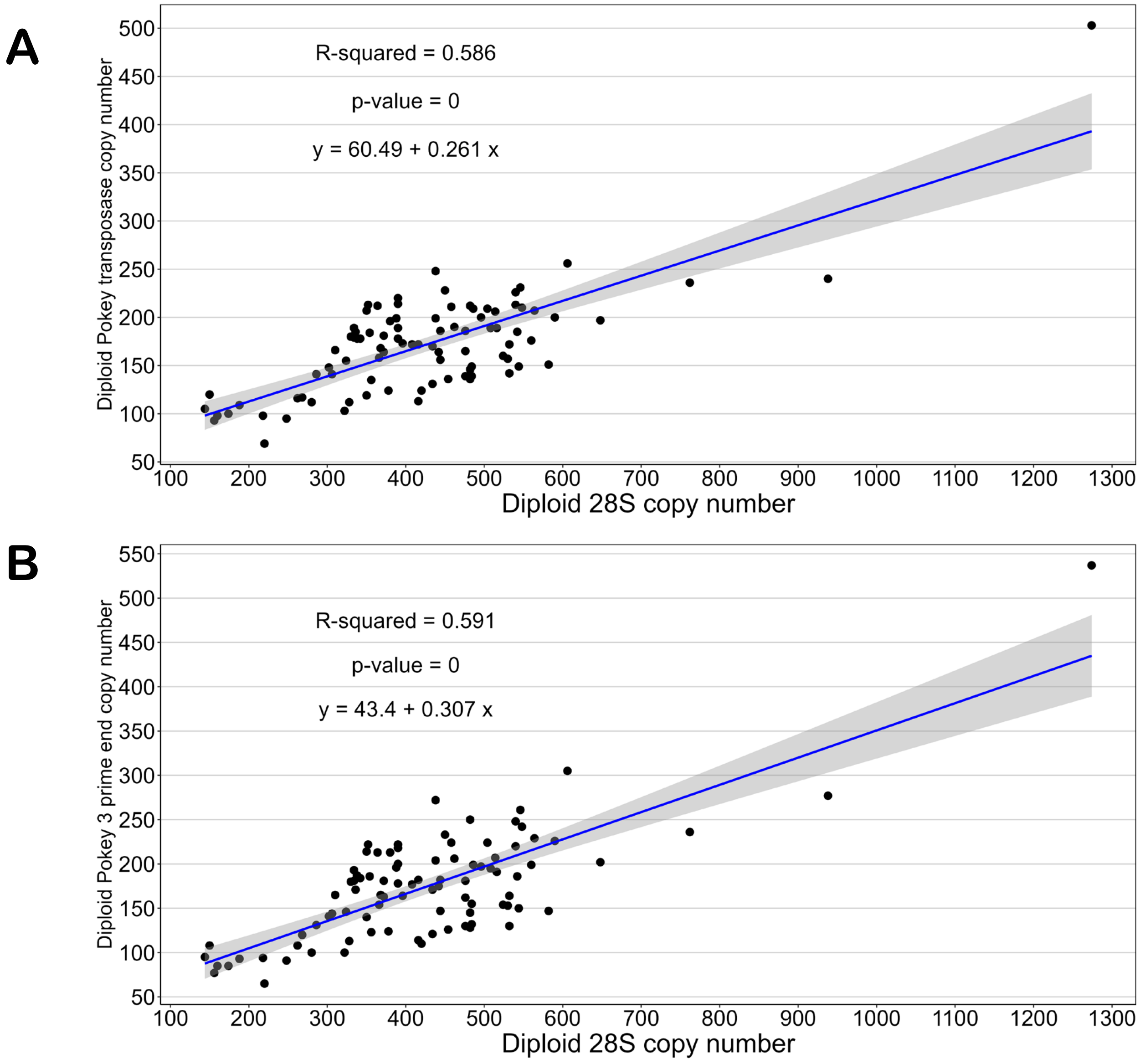

Figure S7. Regression analysis of the relationship between diploid 28S copy number and diploid *Pokey* copy number in 90 samples from *Daphnia obtusa* MA lines. **A.** *Pokey* transposase gene. **B.** Pokey 3′ end.

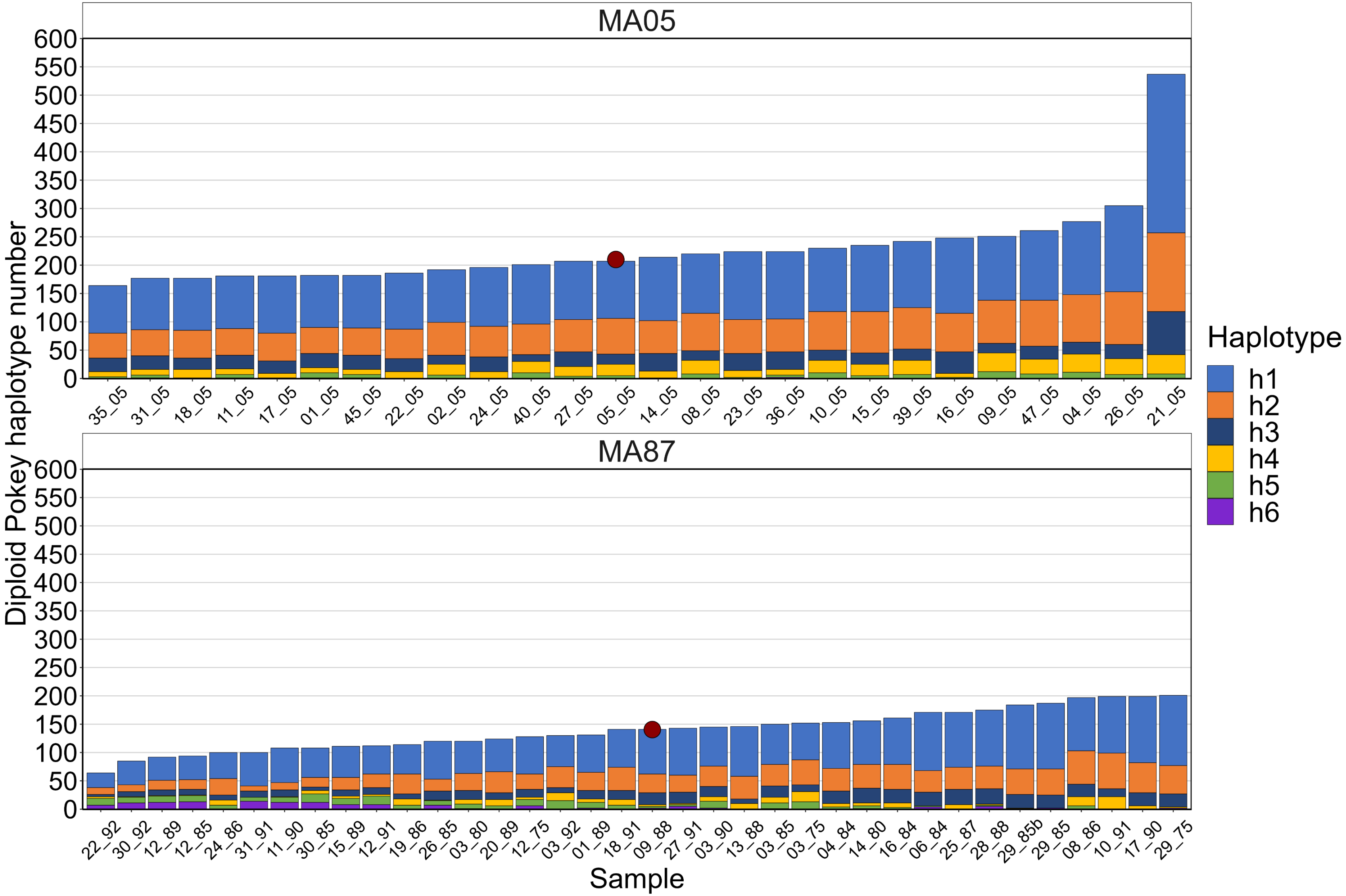

Figure S8. Distribution of six *Pokey* haplotypes in the rDNA of *Daphnia obtusa* MA lines sampled at generation 5 (MA05) and generation ~87 (MA87). The red dot indicates the median copy number for each group. Total diploid *Pokey* copy number is based on the sequence of the *Pokey* 3′ end and the samples are ordered according to the total copy number.

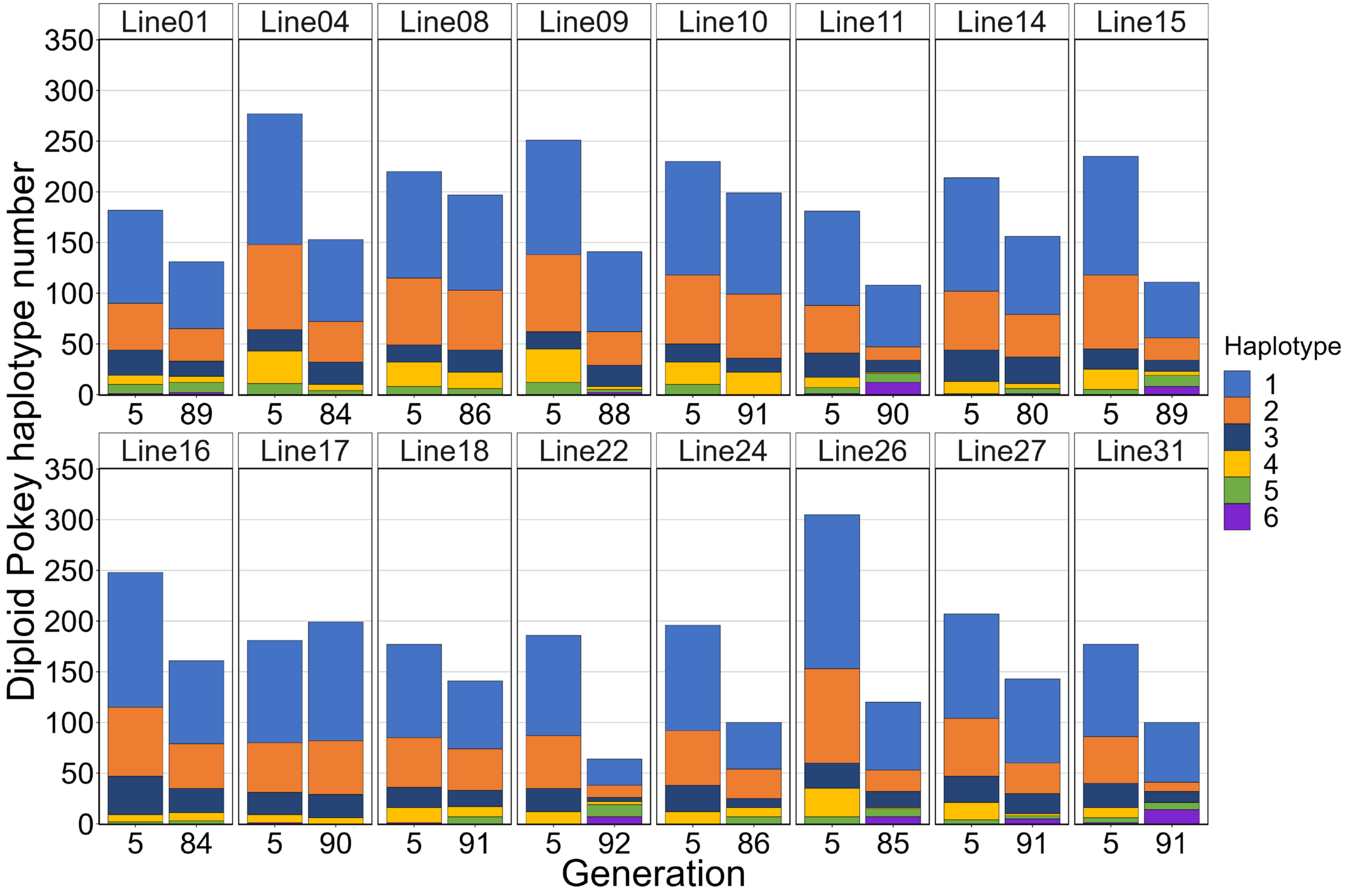

Figure S9. Distribution of six *Pokey* haplotypes in the rDNA of 16 *Daphnia obtusa* MA lines sampled at both generation 5 and generation ~87 (Paired lines). Total diploid *Pokey* copy number is based on the sequence of the *Pokey* 3′ end.

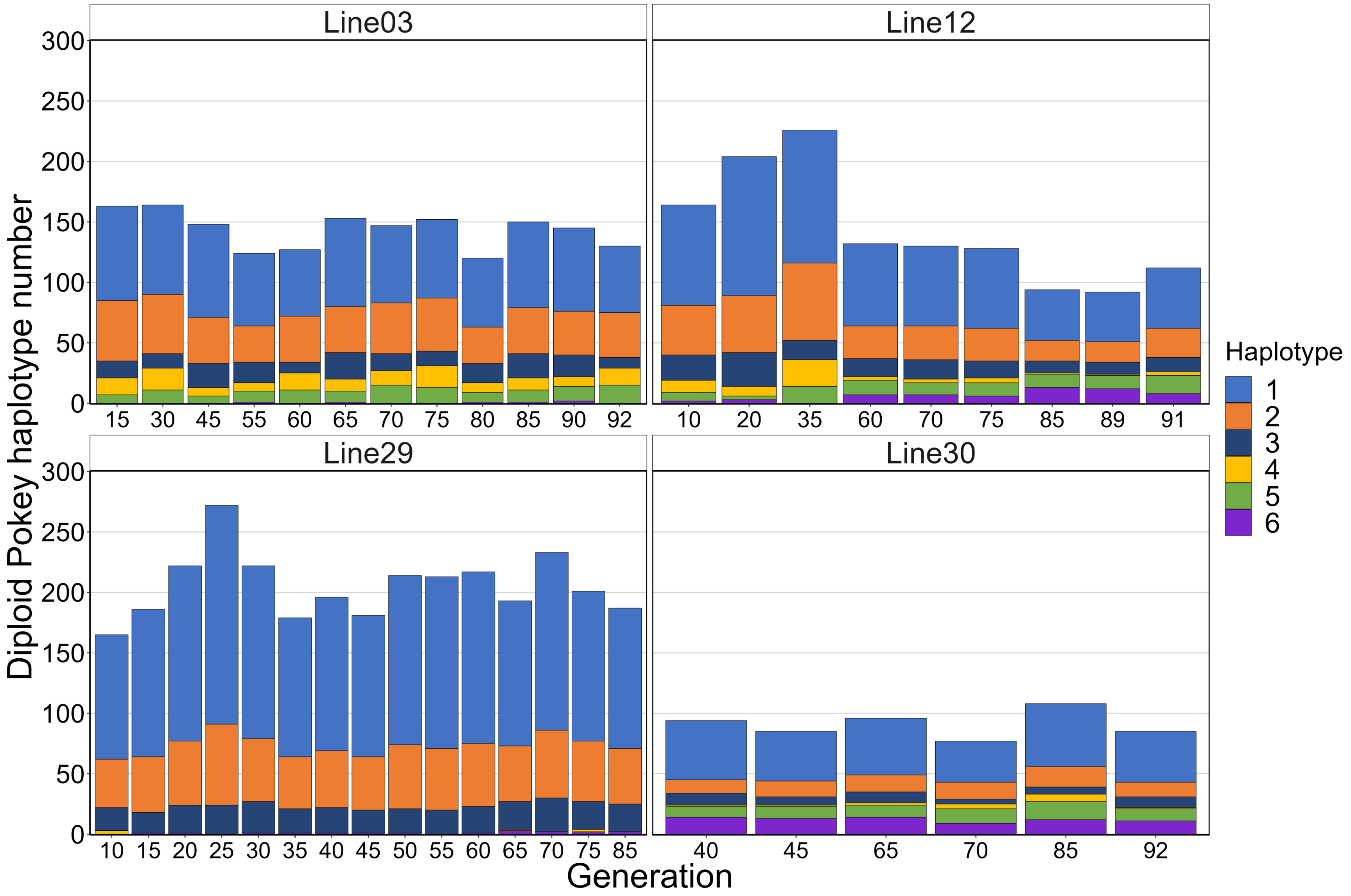

Figure S10. Distribution of six *Pokey* haplotypes in samples from the four fine-grained MA lines of *Daphnia obtusa*. Total diploid *Pokey* copy number is based on the sequence of the *Pokey* 3′ end.

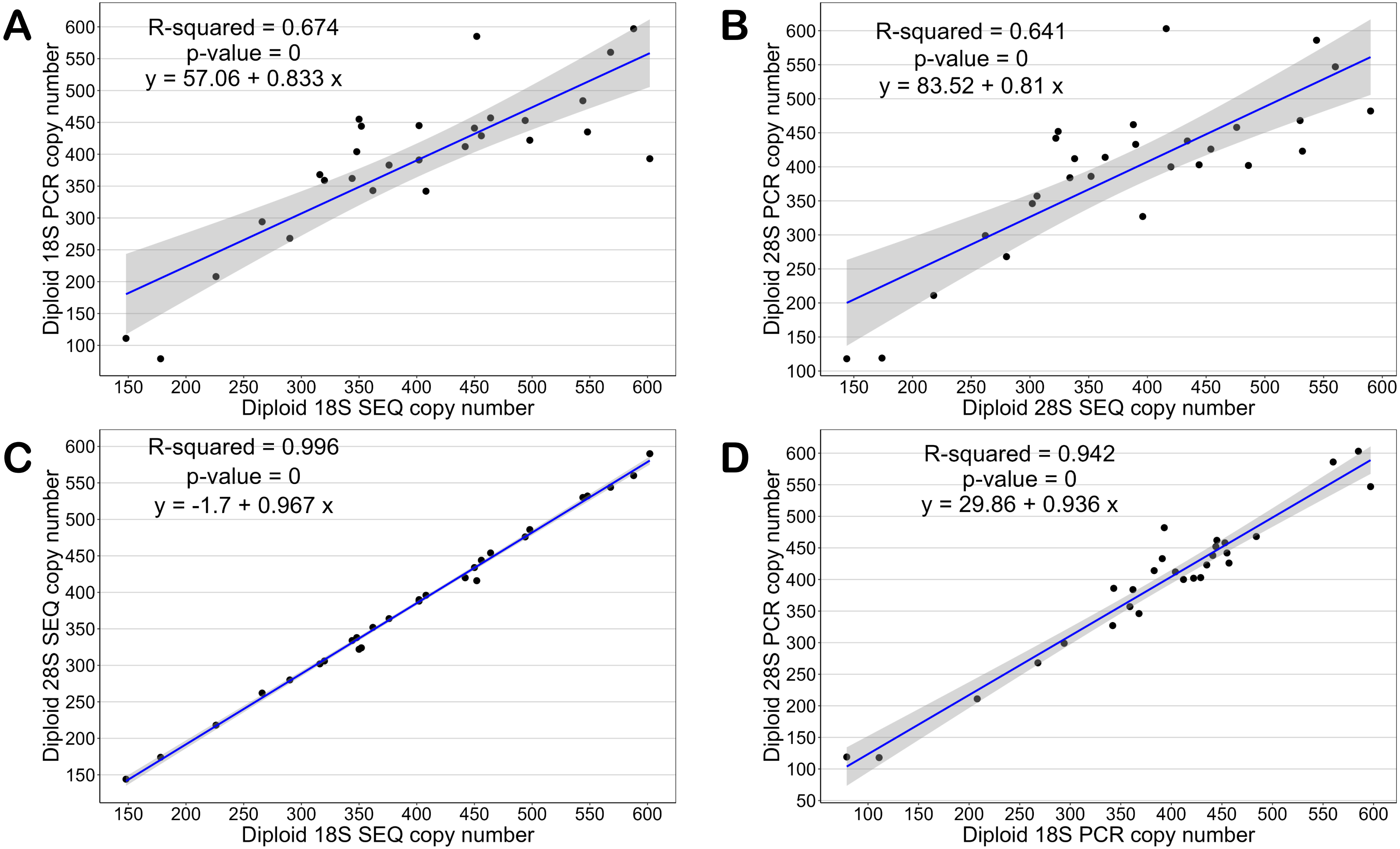

Figure S11. Regression analysis of the relationship between diploid 18S and 28S copy number based on short-read sequencing and qPCR in samples from *Daphnia obtusa* MA lines. The qPCR data are from Leriche et al. (2014). **A.** 18S copy number based on sequencing and qPCR. **B.** 28S copy number based on sequencing and qPCR. **C.** 18S and 28S copy number based on sequencing. **D.** 18S and 28S copy number based on qPCR.

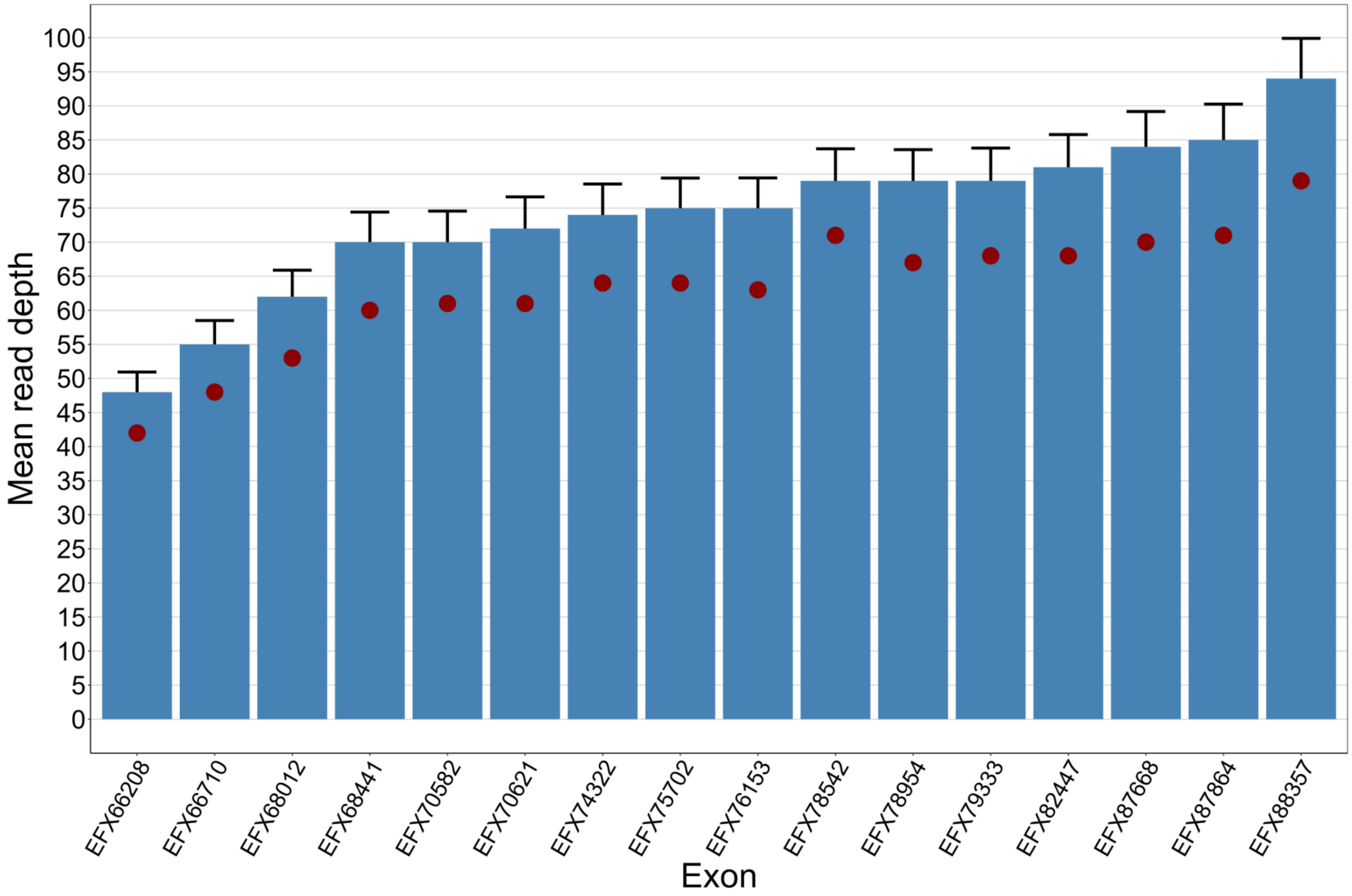

Figure S12. Mean (blue bars) and median (red dots) read depth of the 16 exons used to estimate rDNA and Pokey copy number in 91 genome sequences from the *Daphnia obtusa* mutation accumulation lines. The vertical lines are standard errors.
